# Porous N‐Doped Carbon‐encapsulated Iron as Novel Catalyst Architecture for the Electrocatalytic Hydrogenation of Benzaldehyde

**DOI:** 10.1002/cssc.202400546

**Published:** 2024-10-17

**Authors:** Filippo Pota, Maida A. Costa de Oliveira, Christian Schröder, Marc Brunet Cabré, Hugo Nolan, Aran Rafferty, Olivier Jeannin, Franck Camerel, James A. Behan, Frédéric Barrière, Paula E. Colavita

**Affiliations:** ^1^ School of Chemistry Trinity College Dublin, College Green Dublin 2 Ireland; ^2^ Univ Rennes, CNRS, Institut des Sciences Chimiques de Rennes – UMR 6226 F-35000 Rennes France

**Keywords:** electrocatalysis, electrocatalytic hydrogenation, benzaldehyde, biomass, sustainable chemistry

## Abstract

Carbon porous materials containing nitrogen functionalities and encapsulated iron‐based active sites have been suggested as electrocatalysts for energy conversion, however their applications to the hydrogenation of organic substrates via electrocatalytic hydrogenation (ECH) remain unexplored. Herein, we report on a Fe@C:N material synthesized with an adapted annealing procedure and tested as electrocatalyst for the hydrogenation of benzaldehyde. Using different concentrations of the organic, and electrolysis coupled to gas chromatography experiments, we demonstrate that it is possible to use such architectures for the ECH of unsaturated organics. Potential control experiments show that ECH faradaic efficiencies >70 % are possible in acid electrolytes, while maintaining selectivity for the alcohol over the pinacol dimerization product. Estimates of product formation rates and turnover frequency (TOF) values suggest that these carbon‐encapsulated architectures can achieve competitive performance in acid electrolytes relative to both base and precious metal electrodes.

## Graphical abstract

Porous N‐doped carbon‐encapsulated iron carbide/nitride nanocomposites with good performance towards electrocatalytic hydrogenation of organics were synthesized. Faradaic efficiencies >70 % in acid electrolytes were achieved while maintaining high selectivity for the alcohol product. Performances are competitive vs base and precious metal catalysts, as shown by product formation rates and turnover frequencies.

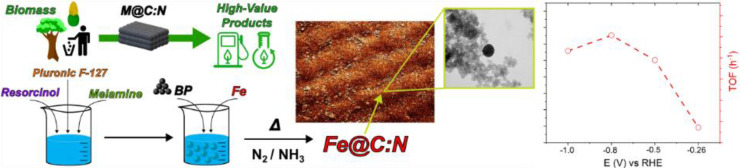

## 1 Introduction

Among various explored strategies to address the increasing demand for sustainable energy, the valorization of biomass and waste feedstocks emerges as a significant alternative to produce fuels, chemicals and for reducing carbon dioxide emissions.[^1^, ^2^] Biomass valorization is highlighted as a valuable approach to mitigate global warming, and its derivatives are seen as promising renewable and abundant hydrocarbon resources with potential for different applications.^[3]^ The main strategies to convert biomass feedstocks to high‐value products are based on hydrogenation processes. Thermal catalytic hydrogenation (TCH) can be an effective method however it requires high temperature, high pressure and high purity H_2_ conditions, and usually the use of precious metal catalysts.^[4]^ An alternative approach is electrocatalytic hydrogenation (ECH), an electrochemical method that can bypass the problem of activating H_2_, thanks to in situ generation of adsorbed hydrogen (H_ads_) via electroreduction of protons/water.^[5]^ ECH can be further driven by renewable electricity, thus making it potentially an attractive alternative route to conventional TCH.[^6^, ^7^]

**Figure 1:**
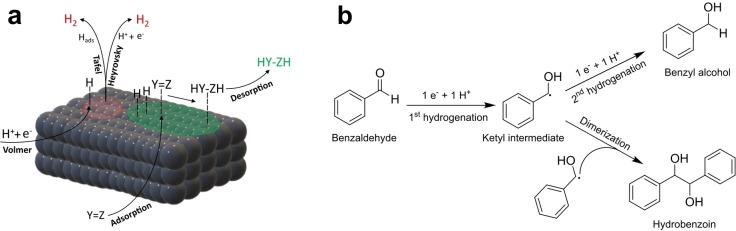
(a) Schematic representation of mechanisms for HER (highlight in red) and ECH (highlight in green). Y=Z is the target organic molecule. (b) Proposed mechanism for benzaldehyde hydrogenation.^[14]^

To realize the potential advantages of ECH, electrocatalysts with good performance in terms of faradaic efficiency (FE), yield and selectivity are needed. FE is typically limited by competition with the hydrogen evolution reaction (HER),[^8^, ^9^] as shown in Figure 1a. The main difference between the HER and ECH is how the H_ads_ is used: Tafel and Heyrovsky steps consume electrons and H_ads_ species and may drastically suppress the FE of organic hydrogenations.^[10]^ Therefore, electrocatalysts must be materials capable of facilitating the Volmer step but that also inhibit the H_2_ evolution pathways.

Several electrocatalyst materials have been proposed as good candidates for the hydrogenation of organic substrates.[^11^, ^12^] Precious metals like Pd, Pt, Au[^13^, ^14^, ^15^] and carbon‐supported Pt‐group metals (M/C) such as Pt/C, Pd/C, and Ru/C[^16^, ^17^, ^18^] have been reported as good electrocatalysts for the hydrogenation of aldehydes and phenols. Despite these encouraging results, the use of precious and/or critical metals is not desirable at scale, particularly when using biomass derived feeds that often vary in composition and purity. For this reason, there is interest in developing abundant and low‐cost electrocatalysts as a suitable replacement. Ni and Cu have been reported as base metals that can either operate as the active phase or that can be alloyed to modulate the activity of precious metals.[^19^, ^20^] Iron‐based catalysts on the other hand have received less attention, despite this being a highly abundant and sustainable metal that has a relatively uniform geographic distribution and therefore a low criticality index.[^21^, ^22^] HMF and glucose hydrogenations were studied by Kwon et al.[^23^, ^24^, ^25^] at Fe electrodes; crotonaldehyde and hydroxyacetone hydrogenations were reported on Fe catalysts;[^26^, ^27^] while Fe supported on graphite was used in the hydrogenation of acetophenone.^[28]^ Furfural was hydrogenated on Fe electrodes with high selectivity in acid electrolyte towards the pinacol dimerization product;^[29]^ however ECH experiments with Fe cathode reported methylfuran as the preferred product.^[30]^ Most recently, an iron‐molybdenum phosphide heterostructured electrode was demonstrated for the electrocatalytic reduction of nitro groups.^[31]^

In this work we discuss the synthesis and study of N‐doped Fe‐carbon heterostructured materials for electrocatalyst applications in the ECH, where the Fe serves as a metal core and the N‐doped carbon matrix as a porous conductive phase in which the Fe sites are embedded (Fe@C:N). Applications of Fe@C:N heterostructures remain largely unexplored in the ECH, despite their use for the electrocatalysis of other reactions,^[32]^ including the ORR^[33]^ and the HER.^[34]^ Electrocatalytic activity was studied via voltammetry and electrolysis experiments aqueous electrolyte using benzaldehyde (BZH) as a diagnostic substrate.^[35]^ Benzaldehyde, due to its good solubility in water^[36]^ and its moderate reactivity,^[35]^ stands out among various potential organic derivatives of biomass as a relevant candidate for studying the reactivity of new electrocatalyst materials for ECH. Importantly, BZH and substituted BZH are commonly found in the pyrolysis of lignin[^37^, ^38^, ^39^, ^40^, ^41^] and are important representatives of biomass‐derived aromatic aldehydes.^[19]^ This justifies the recent interest in BZH, which has become the subject of intense investigation in several studies with encouraging results.[^42^, ^43^, ^44^] The ECH of benzaldehyde has been previously investigated using Pt‐group electrocatalysts and is reported to result in formation of benzyl alcohol and/or in dimerization to hydrobenzoin, as in Figure 1b.^[14]^ Both of these products are of interest: benzyl alcohol is the most important aromatic alcohol from a commercial perspective and a high value commodity with applications in e. g. coatings, plastics, cosmetics and food;[^45^, ^46^] while hydrobenzoin is a high‐value compound with applications in the pharmaceutical industry.[^19^, ^47^] Achieving ECH with high faradaic efficiency and good selectivity towards one of these two products using Fe@C:N heterostructures is therefore of interest both from a mechanistic and an application perspective. Our results show that this is indeed possible with such electrocatalyst architectures thanks to potential control. A comparison of turnover frequency (TOF) estimates obtained from our results suggest that these materials could be a competitive alternative for the ECH of bio‐derived organics.

## 2 Experimental Section

### 2.1 Materials

Iron (II) acetate (95 %), Nafion^®^ 117 solution (5 %), resorcinol (99 %), Pluronic F‐127, melamine (99 %), hydrochloric acid (37 %), sulfuric acid (95–98 %), nitric acid (≥65 %), formaldehyde solution (37 wt %), hydrogen peroxide (30 %), sodium sulphate (>99 %); benzaldehyde (BZH, >99 %); hydrobenzoin (HBZ, 99 %); acetophenone (ACP, >99 %); ethyl acetate (>99 %) were all purchased from Sigma Aldrich and used as received. Ethanol (>99 %), benzyl alcohol (BA, >99 %) were purchased from Fisher and used as received. Black Pearls 2000^®^ (BP, *ca*. 1400 m^2^/g)^[48]^ were purchased from Cabot.

### 2.2 Catalyst Synthesis

BP was first pre‐treated via oxidative purification to eliminate metal impurities and enhance hydrophilicity.[^49^, ^50^] The material was subjected to a 4 h reflux in concentrated HNO_3_ at 90 °C, followed by filtration and washing with distilled water until a neutral pH was achieved. The resulting carbon paste was then dried for two days at 50 °C and subsequently ground in an agate mortar prior to further use.[^33^, ^51^] Porous carbon‐based catalysts were prepared as follows: resorcinol (1.65 g), Pluronic F‐127 (2.5 g), and melamine (0.84 g) were first dissolved in a 40 mL solution of water:ethanol (1 : 1 by vol.) and stirred for 15 min. Fe(CH_3_COO)_2_ (1.5 g) was added to this mixture as a metal precursor, then concentrated HCl (0.2 g), followed by stirring for 1 h. Formaldehyde solution (2.5 g) was added dropwise under stirring, and the mixture was stirred vigorously for another hour, yielding a homogeneous slurry. Purified BP (1.5 g) was added to this dispersion and stirred for an additional 15 min. The mixture was heated in an oven at 50 °C for 2 days in a Teflon‐lined autoclave. Samples were dried for two days at 50 °C, ground in a mortar and further dried in a tube furnace (250 °C) under N_2_ flow. Finally, the product was annealed (800 °C, 2 h) in a N_2_:NH_3_ flow (1 : 1 vol., 200 sccm) followed by cooling under N_2_, as show in Figure S1.

### 2.3 Materials Characterization

Brunauer–Emmett–Teller (BET)^[52]^ specific surface area was determined from nitrogen adsorption at 77 K with a Nova 4200e Surface Area Analyzer (Quantachrome, UK), with the specific surface area calculated from data at relative pressures between 0.10 and 0.30. Pore size distributions were calculated using the Barrett–Joyner–Halenda (BJH) method^[53]^ from the desorption branch of the isotherm. Prior to analysis, the sample was outgassed at 250 °C under vacuum for 2 h. Small angle X‐ray scattering (SAXS) was carried out using a custom‐built setup with a Cu microfocused generator (GeniX‐3D VHF‐L, Xenocs) at 50 W, two slit (JJ X‐ray) collimation and an image plate detector (Mar345, MarResearch). Thermogravimetric analysis (TGA) was carried out using a Pyris 1 Thermogravimetric Analyzer (PerkinElmer) with a hold time of 10 min at 150 °C, followed by a 10 °C/min ramp in air to 900 °C. Fourier transform infrared spectroscopy (FTIR) was carried out on a FTIR Spectrum 100 spectrometer (PerkinElmer) using an attenuated total internal reflectance (ATR) diamond crystal; 4 scans between the ranges of 4000 cm^−1^ and 400 cm^−1^ were collected for each sample and ratioed against background spectra obtained in air. Powder X‐ray diffraction (XRD) was carried out to investigate the crystalline structures before and after annealing using a D2 Phaser diffractometer with LynxEye detector (Bruker) and a Cu Kα source. Scanning electron microscopy (SEM) was performed using a Zeiss Ultra microscope at 5 kV accelerating voltage, and InLens detector. Transmission electron microscopy (TEM) was performed using a Jeol JEM 2100HR with an acceleration voltage of 200 kV, EDS SDD Oxford X–Max 80T detector and Jeol HAADF detector. Inductively coupled plasma‐optical emission spectroscopy was performed in a iCAP 7000 ICP‐OES Analyser (Thermo Fisher); samples were treated in HNO_3_ (≥65 %) overnight and diluted in Millipore water to a nominal concentration of 10 mg/L. Raman spectroscopy was carried out using a Witec alpha 300R confocal scanning Raman system using 532 nm excitation; peak areas were analyzed using commercial software (CasaXPS). X‐ray photoelectron spectroscopy (XPS) was carried out in a monochromated Omicron XM1000MK II X‐ray photoemission spectrometer, with an EA 125 analyzer and an Al Kα (1486.7 eV) source; pass energies of 50 and 15 eV were used for survey and high‐resolution spectra, respectively. Spectra were analyzed using CasaXPS; best fits were carried out on spectra after Shirley background correction using mixed Gaussian‐Lorentzian functions. Elemental compositions were obtained from peak areas of the high‐resolution scans. Product analysis was carried out using an Agilent 8860 Gas Chromatograph coupled to a flame ionization detector (GC‐FID), H_2_ as carrier gas, split injection (1 : 10, 2 mL/min flow) and a DB‐WAX UI column (30 m×0.250 mm×0.50 μm, Agilent J&W).

### 2.4 Electrochemical Characterization

Catalyst (0.0100 g±0.0003) was used for the preparation of an ink dispersion, adding 135 μL of Millipore water, 270 μL of isopropyl alcohol (IPA) and 50 μL of Nafion solution. The ink was sonicated at room temperature prior to drop casting on carbon working electrodes. Glassy carbon disks (GC, Ø 5 mm, HTW GmbH) were used as working electrodes for cyclic voltammetry experiments. GC disks were polished with decreasing grades of alumina slurry (Ted Pella) according to published procedures;[^54^, ^55^] disks were thoroughly sonicated and rinsed between polishing steps to remove any residue from the preceding step. GC disks were mounted in a Teflon disc holder (Pine instruments) and modified with the ink dispersion (10 μL), then dried under inert gas, resulting in a loading of 1.10 mg cm^−2^. A Pt disk electrode (Ø 3 mm, eDAQ) was used for comparison; prior to experiments using Pt as electrode, the disk was polished and then cleaned by cycling into the anodic region until the characteristic response of polycrystalline Pt was obtained.[^56^, ^57^, ^58^] Flags of carbon cloth (CC, 1 cm^2^ geometric area, FuelCellStore) with microporous layer were used as working electrodes for electrolysis experiments. The CC was used as received after covering the back side with insulating polyimide tape. 50 μL of the ink were drop cast in two equal aliquots, drying the ink after each step in a hot plate (80 °C) followed by compression of the CC in a hydraulic press (Specac) at room temperature; this resulted in loadings of 1.09 mg cm^−2^ for all experiments.

Cyclic voltammetry experiments (CV) were carried out using a standard 3‐electrode cell controlled by a potentiostat (Metrohm Autolab), using graphite rods (Morgan Advanced Materials) as counter electrodes (CE) and Ag/AgCl (1 M KCl) as reference electrode (RE, 0.235 V vs SHE). All potentials are reported vs the relative hydrogen electrode (RHE) based on the experimentally determined pH of the electrolyte. The cell was cleaned with piranha and Millipore water prior to each experiment, then filled with 250 mL volume of supporting electrolyte (0.100 M H_2_SO_4_); the electrolyte was purged with N_2_ for at least 15 min prior to characterization. Estimates of electrochemically active surface area (ECSA) were obtained from capacitive CV curves in Na_2_SO_4_ 0.100 M over a no‐Faradaic potential window; the double layer capacitance was normalized by an average value of 40 μF cm^−2^ to yield estimates of ECSA.[^56^, ^59^] Electrolysis was performed in an H‐cell, with compartments separated by an ion‐conducting membrane (FKE‐50 Fumasep) to minimize cross contamination (Figure S2). WE and RE were inserted in 34 mL of catholyte (BZH in 0.1 M H_2_SO_4_), and the CE was inserted in 34 mL of anolyte (0.1 M H_2_SO_4_). Before starting the electrolysis both compartments were purged with N_2_ for at least 15 min; 2 mL of catholyte were aliquoted for quantitative analysis at *t*_0_ *=*0 s. CVs at 100 mV/s and 10 mV/s were first collected to condition the electrode surface, followed by measurement of the iR drop via the i‐interrupt method (NOVA). Potentiostatic electrolysis was carried out for 2 h, followed by analysis via GC‐FID of a 2 mL aliquot of the catholyte; the catholyte was extracted in ethyl acetate (2 mL, 99.1 % and 89.9 % efficiency for BZH and BA, respectively) adding ACP as internal standard.

## 3 Results and Discussion

**Figure 2:**
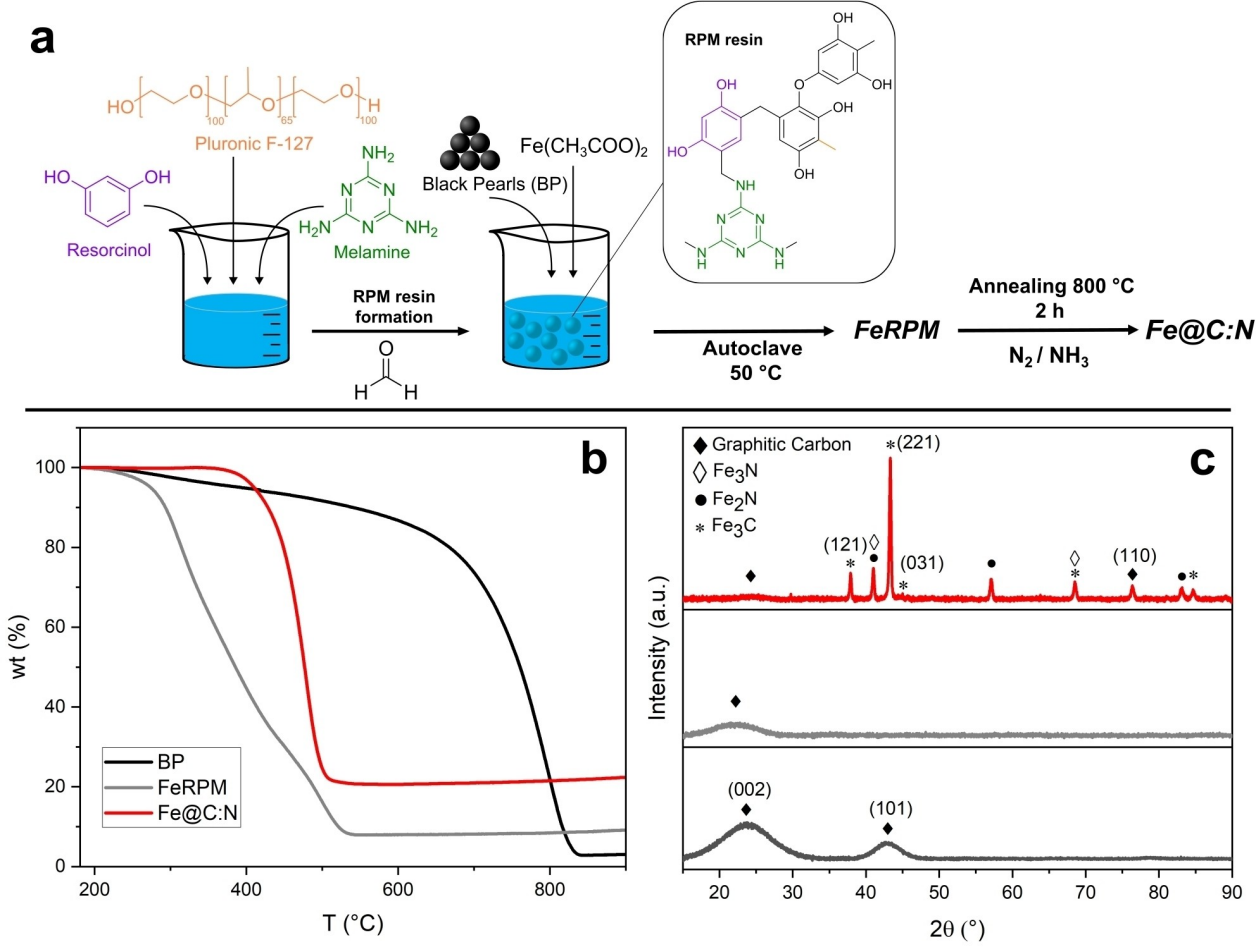
(a) Scheme showing the main steps in the materials synthesis protocol used in this work. (b) TGA curves obtained in air at 10 °C/min for materials FeRPM, Fe@C:N and the carbon black support BP. (c) XRD patterns of the same carbon‐based materials; characteristic peaks are indicated with assignments.

Electrode materials for HER and ECH studies were synthesized using a protocol reported by our group,^[33]^ but adapted via modification of the annealing process to include a pre‐annealing step at high temperature under N_2_ flow. Briefly, the synthesis was based on hydrothermal reactions of a resorcinol resin^[60]^ in the presence of metal precursors, a soft template and a conductive carbon black, as schematically shown in Figure 2a. Resorcinol resins are synthesized via polycondensation of resorcinol and formaldehyde in a mixture of alcohol and water containing BP as conductive carbon support. The presence of melamine and Pluronic F‐127 leads to nitrogen incorporation in the carbon network and increases the surface area of the material,[^61^, ^62^] yielding a BP‐supported resin here onwards referred to as FeRPM. The FeRPM resin was then pyrolyzed at high temperature under N_2_/NH_3_ flow using a new thermal annealing protocol in Figure S1, to graphitize the carbon matrix surrounding the N‐functionalities and Fe‐rich clusters, thus yielding the material under investigation referred to as Fe@C:N.

The Fe@C:N materials were found to have a porous structure with specific surface area of 430 m^2^/g determined from N_2_ adsorption isotherms (Figure S3). The BJH pore distribution shows that the material is mesoporous, as expected when using Pluronic‐F127 as a soft template[^63^, ^64^] and in agreement with our previous results on similar materials.^[33]^ SAXS characterization, however, did not show evidence of an ordered mesostructure (Figure S4) as reported using other resorcinol‐pluronic resin protocols;[^65^, ^66^, ^67^] this is likely due to the incorporation of iron precursors and/or the conductive BP scaffold during the first hydrothermal pyrolysis step.

Bulk composition of Fe@C:N material was investigated via ICP‐OES yielding an Fe‐content of 10.7 % by wt. Figure 2b shows TGA curves in air for FeRPM and Fe@C:N samples, i. e. before and after the annealing process; data for the BP scaffold materials is also shown for comparison. Pure BP particles undergo a single combustion process at high temperature yielding a negligible residual mass of 3.7 %. The FeRPM solid obtained from the hydrothermal reaction yielded two broad weight loss processes, a first one at *ca*. 230 °C and a second one at *ca*. 430 °C, with a final residual mass of 9.47 %. After graphitization, Fe@C:N materials show a single well defined mass loss at *ca*. 360 °C with a final residue of 20.6 %. This observation is consistent with the sample undergoing graphitization, yielding a more crystalline and stable carbon structure that is oxidized at higher temperatures than the partially graphitized FeRPM. Assuming that the inorganic residue from Fe@C:N consists of Fe_2_O_3_ exclusively, the observed 20.6 % corresponds to an estimated Fe‐content of 14.4 % by wt. which is in excellent agreement with values determined using ICP‐OES, after accounting for the BP solid residue. Notably, a lower decomposition temperature was observed for Fe@C:N compared to BP; this is likely due to the presence of iron oxides which are known to catalyze the oxidation of carbons.^[68]^ FTIR transmittance spectra for FeRPM and Fe@C:N further supported TGA results indicating that the material undergoes significant graphitization (see Figure S5). The FeRPM spectrum shows broad peaks consistent with the presence of an RPM resin,[^62^, ^69^] while the absence of peaks in the spectrum of Fe@C:N is supportive of decomposition of the organic precursors and concomitant graphitization. Finally, Raman spectra also support graphitization as evidenced by an increase after annealing of the D‐band contribution that is associated with the breathing mode of six‐membered rings in graphitic carbons (Figure S6), and in agreement with graphitization trends in amorphous carbon materials.^[70]^

Figure 2c shows XRD data for BP, FeRPM and Fe@C:N materials. The only prominent peaks in the patterns of BP and FeRPM can be indexed as (002) (at 23.7°, d=3.75 Å) and (101) (at 42.9°, d=2.10 Å) reflections of the graphite structure, in agreement with literature values.[^65^, ^71^] The large fwhm observed is indicative of structural disorder in the carbon phase. In the case of Fe@C:N the pattern is instead dominated by relatively narrow and intense peaks; reflections at 2θ=37.8°, 43.3° and 44.5° are in good agreement with (121), (211) and (031) reflections, respectively, of Fe_3_C;^[33]^ peaks at 2θ=41.0°, 57.1°, 68.5°, 83.0° and 84.7° are assigned to contributions from nitrides and carbides in agreement with reference patterns for Fe_2_N (PDF#73‐2102), Fe_3_N (PDF#01‐1236) and Fe_3_C (PDF#89‐2005). The formation of nitrides and carbides is consistent with a process involving carbonization of the organic resin under the ammonia‐rich atmosphere while in the presence of iron salts. XRD does not show evidence of crystalline Fe^0^, given the absence of any of the characteristic peaks at 65.2° and 85.2°, or at 63.4° and 80.1° of metallic iron patterns PDF#87‐0722 and PDF#89‐4186, respectively (Figure S7). Therefore, the XRD pattern indicates that the annealing process results in the decomposition of the iron salt and its reduction to carbide‐nitride solids.

**Figure 3:**
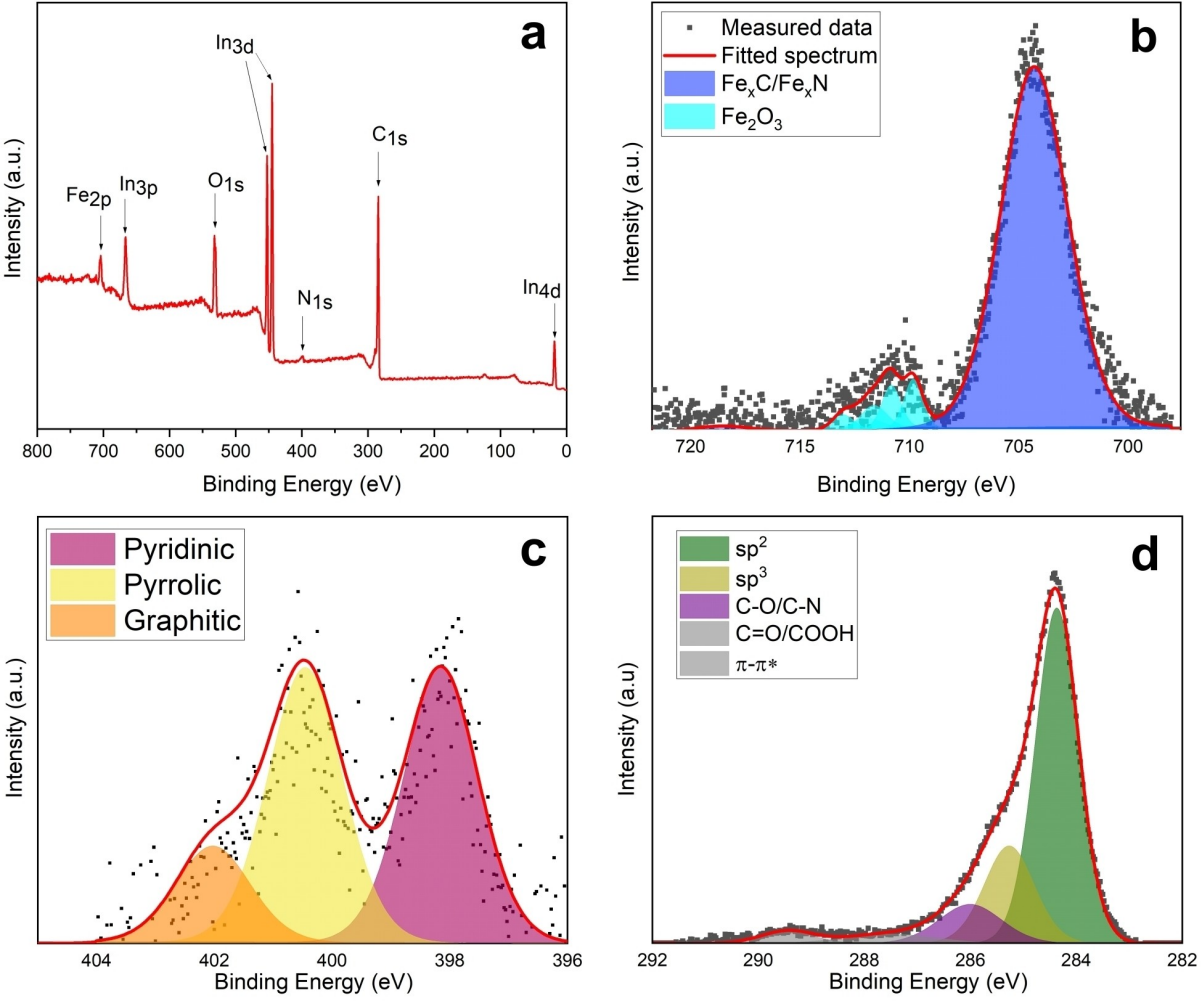
XPS (a) survey, (b) Fe 2p_3/2_, (c) N 1s and (d) C 1s spectra of Fe@C:N; best‐fit components and simulated spectral envelope are also shown where relevant.

Surface composition of annealed Fe@C:N samples was characterized using XPS. Figure 3a shows the survey spectrum displaying characteristic Fe 2p_3/2_ (710 eV), N 1s (398 eV), C 1s (284 eV) and O 1s (531 eV) peaks; additional peaks are due to contributions from the In foil used as support.^[72]^ The elemental composition is reported in Table 1; values were obtained from the area ratios of the high‐resolution scans of the core level excitations, shown in Figures 3b–3d, after correction by relative sensitivity factors. The oxygen content of Fe@C:N was estimated by subtracting the oxide contribution arising from the indium foil (In_2_O_3_), from the total area of the O 1s peak (see Figure S8). Figure 3b shows the best‐fit of the Fe 2p_3/2_ spectrum, which displays two maxima at 704 and 710 eV. The peak at 704 eV was best‐fit using a single component that can be assigned to Fe, Fe_x_C or Fe_x_N species based on its binding energy.[^73^, ^74^, ^75^] Iron carbides, its nitrides and metallic iron all have similar binding energies and cannot be fully resolved via XPS,[^73^, ^74^, ^75^] however, based on the XRD results the contribution at 704 eV likely arises from carbides and/or nitrides. The envelope with maximum at 710 eV is consistent with the presence of Fe(III) oxides/oxyhydroxides, which typically give rise to multiple peaks and an overall asymmetric lineshape;^[76]^ accordingly, the best‐fit was obtained using multiple peaks (709.8, 710.8, 711.6, 711.9 and 713.0 eV) all assigned to Fe(III) oxide species. The 704 eV peak has significantly higher intensity than the oxide components, thus indicating that most of the iron present at the surface/sub‐surface is in a low oxidation state. Importantly, metallic iron and its carbides/nitrides are known to be easily oxidized in air;[^77^, ^78^] therefore, the absence of significant FeO_x_ contributions in the Fe 2p spectrum strongly suggests that iron‐rich phases are encapsulated and thus protected from oxidation by a carbon shell.

**Table 1:** Elemental composition in atomic‐%, obtained from XPS high‐resolution spectra of Fe@C:N.

Material	C (%)	O^[a]^ (%)	N (%)	Fe (%)	N_Graphitic_ (%)	N_Pyridinic_ (%)	N_Pyrrolic_ (%)	$C_{sp^{2}}/C_{tot}$ ^[b]^
Fe@C:N	88.5	7.39	2.25	1.82	14.9	42.5	42.6	60.9

[a] atomic‐% content calculated from the contribution to the O 1s area that does not arise from I_2_O_3_. [b] Calculated as area ratio of the best‐fit component at 284.4 eV over the total C 1s peak.

Figure 3c shows the best‐fit of the N 1s spectrum which was obtained using three components attributed to graphitic‐N (400.9 eV), pyrrolic‐N (400.0 eV) and pyridinic‐N (398.0 eV).[^79^, ^80^] The relative contributions of these three functionalities are reported in Table 1 and indicate that the majority of the nitrogen is present in the form of pyrrolic/pyridinic groups. It was not possible to resolve any contributions at 397 eV expected for Fe–N species;^[75]^ this is likely due to their relative contributions being much smaller than those arising from N‐functionalities in the carbon scaffold. Figure 3 d shows the best‐fit of the C 1s spectrum obtained using five components, attributed to *sp* ^*2*^ (284.4 eV) and *sp* ^*3*^ (285.3 eV) carbon, C−O/C−N (286.0 eV), C=O/COOH (287.5 eV) groups, and π–π* transitions (289.5 eV).[^72^, ^79^, ^81^, ^82^] The absence of a carbide peak indicates that most of the C 1s intensity arises from the graphitized scaffold; this is consistent with a surface Fe content of ca. 1.8 % (Table 1), that indicates that the contribution arising from carbide should account for <0.7 % of the total C 1s peak, based on the Fe_3_C stoichiometry. The *sp* ^*2*^‐C component is larger than the *sp* ^*3*^‐C one, also supporting that the majority of carbon observed is part of a graphitized network as the result of the annealing process. As shown in Figure S9, the $C_{sp^{2}}/C_{tot}$ content increases from 23.7 % to 60.9 % when the FeRPM material is annealed to Fe@C:N, thus supporting the conclusion that the annealing process increases the degree of graphitization in agreement with FTIR and Raman results.

**Figure 4:**
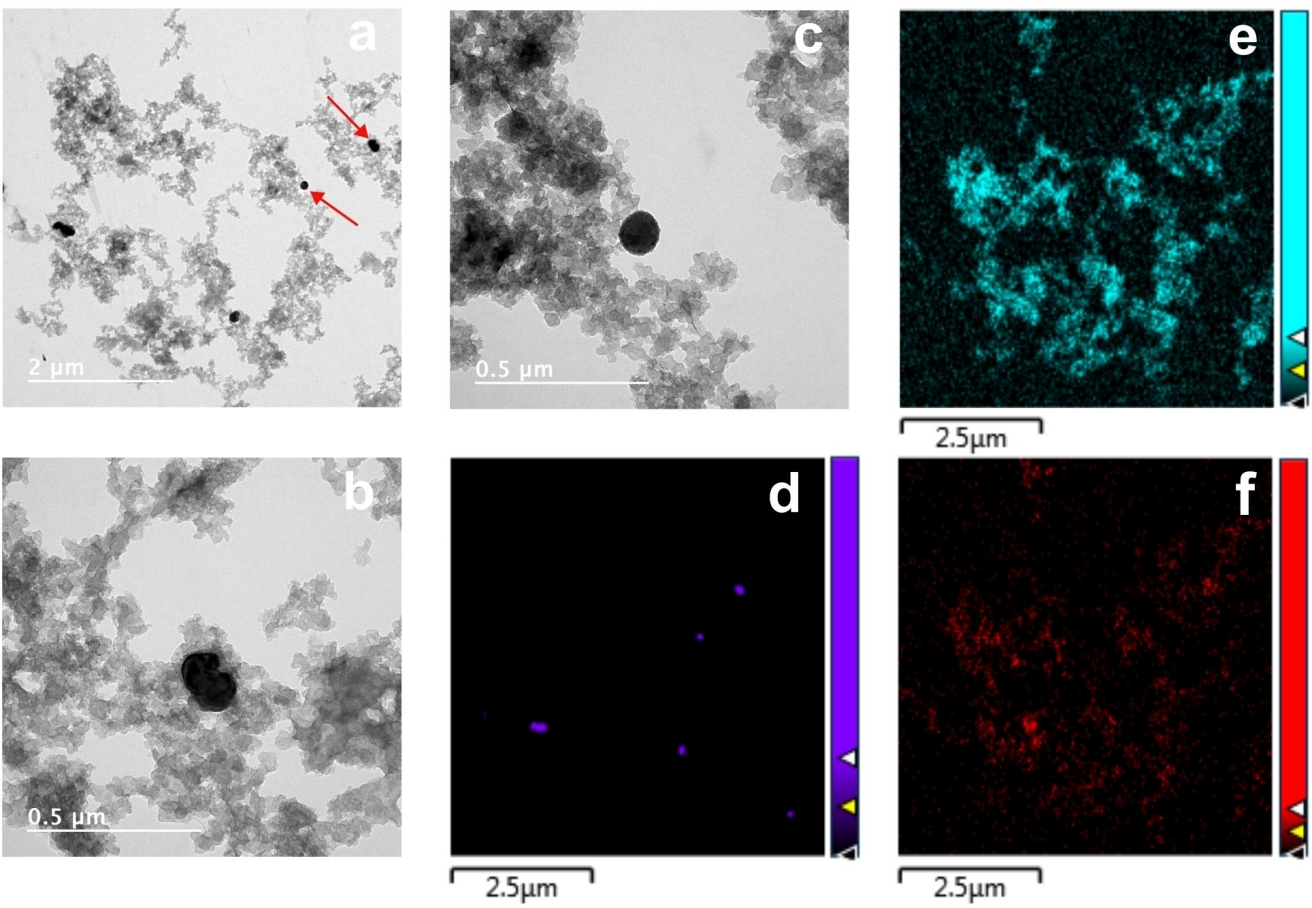
TEM images of (a) Fe@C:N and (b,c) expanded images with details of metal‐rich nanoparticles (indicated with arrows in (a)) resulting from the annealing process. TEM‐EDX chemical mapping of the image in (a) in the (d) Fe, (e) C and (f) O spectral regions.

The morphology of the material was investigated via electron microscopies. Figure S10 shows SEM images of the Fe@C:N solid, which indicate that the sample consists of a complex matrix containing dispersed/embedded nanoparticles characterized by a rough and irregular surface. Further investigation via TEM reveals that the solid consists of nanoparticles with size similar to those of the primary BP particles, as shown in Figures 4a–4c, and additional larger and denser particles with diameter of 150±57 nm. Figures 4d, 4e and 4f show TEM‐EDX images of the Fe, C and O signals, respectively, that show that the larger particles are rich in Fe and are dispersed over a graphitized carbon scaffold support.

**Figure 5:**
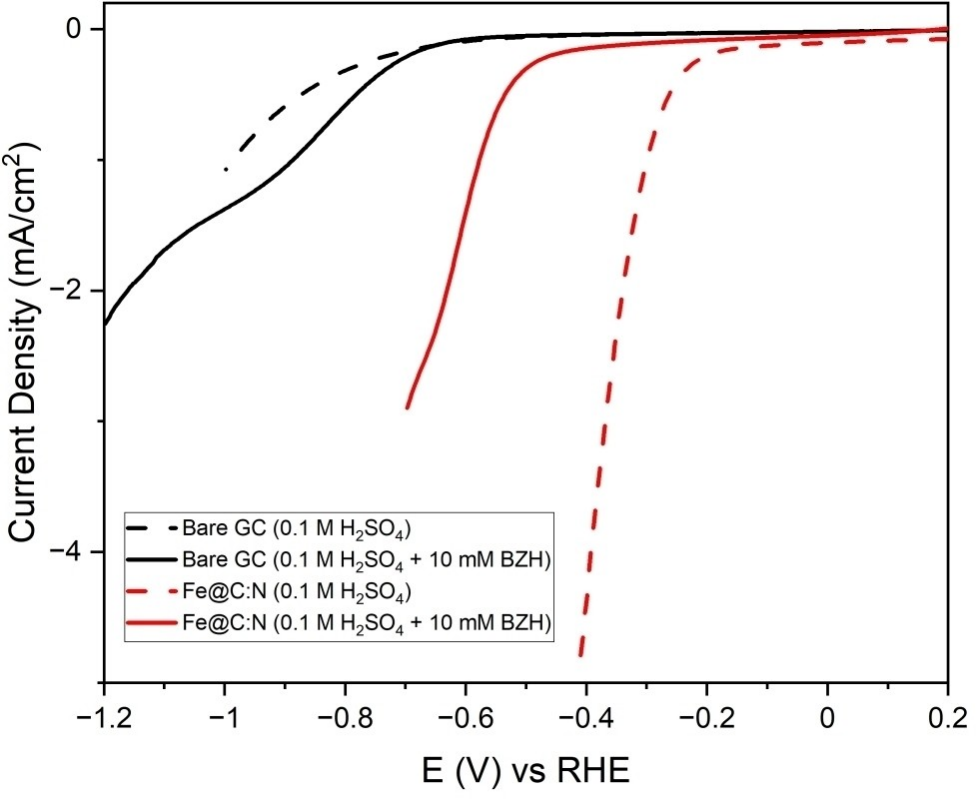
CVs collected for bare CC (black) and Fe@C:N (red) in 0.1 M H_2_SO_4_ (dash line) and H_2_SO_4_ with 10 mM BZH (solid line), at 5 mV/s scan rate in 3 cycles. Only the cathodic region of the last cycle is plotted.

To evaluate the electrochemical response in both HER and ECH reactions, voltammetry experiments were carried out in aqueous electrolytes using BZH as a diagnostic organic compound. Figure 5 shows linear sweep voltammograms (LSV) in the cathodic region obtained for polished GC and Fe@C:N disk electrodes in 0.1 M H_2_SO_4_ at 5 mV s^−1^, in the absence and presence of 10 mM BZH. In background electrolyte, GC displays poor HER activity (η ≅−0.99 V at 1 mA cm^−2^) in agreement with previous findings.^[56]^ Fe@C:N yields a lower overpotential (η≅−0.30 V at 1 mA cm^−2^), which indicates improved HER currents relative to GC albeit below values observed at a Pt disk under the same conditions (see Figure S11).^[56]^ HER activity in the −0.3 to −0.5 V region is attributable to the incorporation of iron carbide/nitride nanoparticles at the surface/subsurface of the electrode material. Indeed, the overpotential at 10 mA cm^−2^, Tafel slope and exchange current density values estimated at Fe@C :N disk electrodes (see Figure S12, Table S1) are in good agreement with those reported in literature for mild steel and cast iron in acid,[^83^, ^84^, ^85^, ^86^] suggesting that HER currents are dominated by Fe/Fe_x_C sites.

In the presence of 10 mM of BZH, bare GC and Fe@C:N show a change in cathodic current densities, with η of −0.89 and −0.57 V (at 1 mA cm^−2^), respectively, observed in the HER region. This indicates that benzaldehyde adsorbs and blocks surface sites involved in proton reduction, quenching the HER activity at Fe@C:N as previously observed in the case of precious metal electrocatalysts[^20^, ^87^] and of base metal electrodes in acid aldehyde solutions.^[23]^ Interestingly, cathodic peaks are observed at *ca*. −0.92 and −0.64 V for GC and Fe@C:N, respectively, associated with BZH reduction.^[13]^ The Pt disk electrode tested under the same conditions does not display an evident reduction peak (Figure S11), however the high HER current background dominates the cathodic response at these surfaces below −0.4 V.

**Figure 6:**
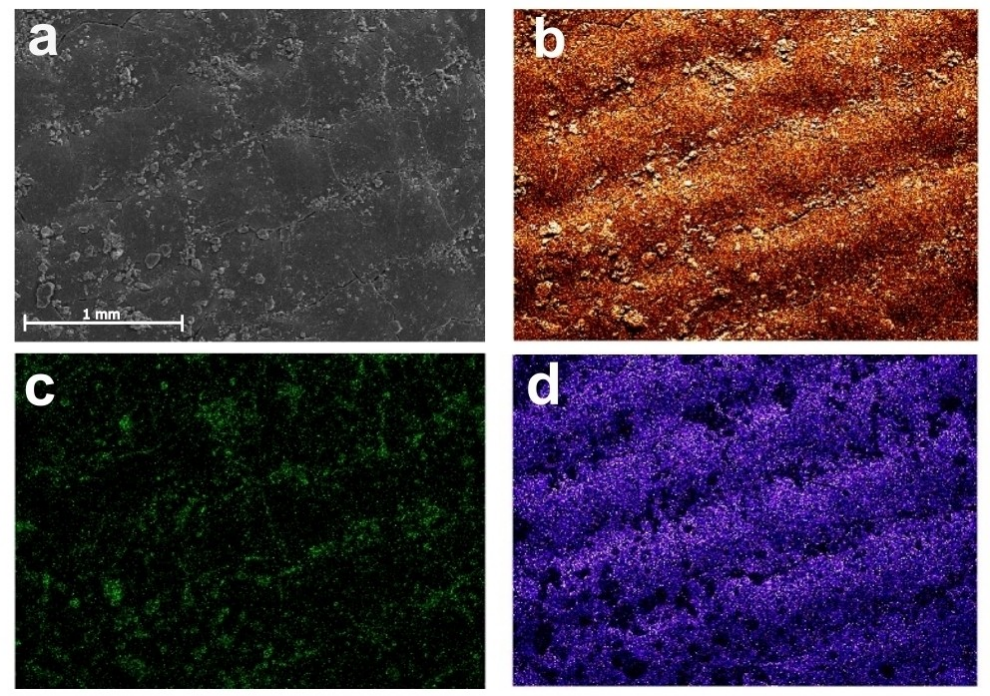
(a) SEM image of a CC electrode decorated with the Fe@C:N electrocatalyst ink, showing the structure of woven fibers in the CC underlying support. SEM‐EDX mapping for C (b), Fe (c) and F (d) atomic species of the electrode surface in (a) indicating uniform distribution of the ink material.

**Figure 7:**
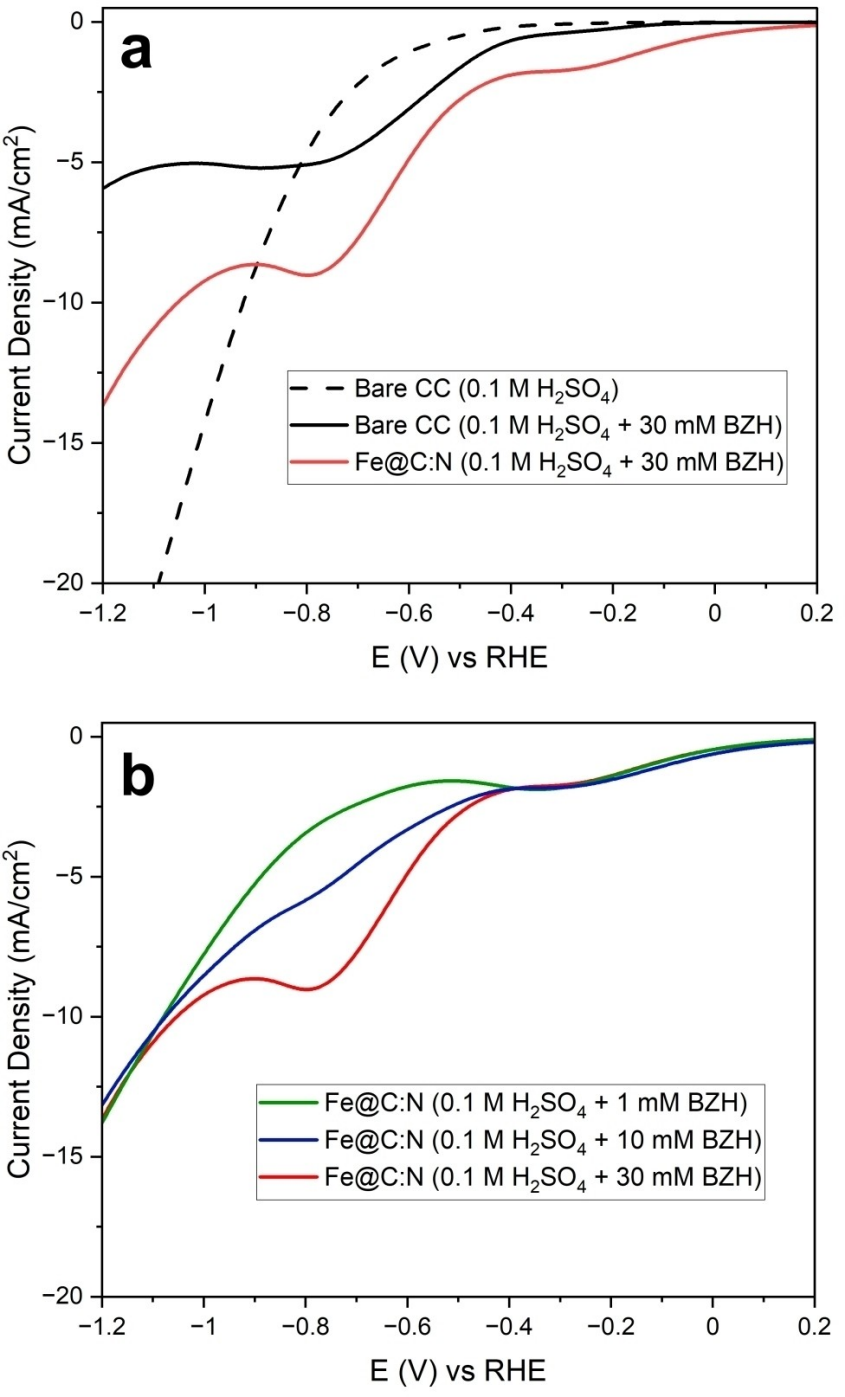
(a) CVs collected for bare CC (black) and Fe@C:N (red) in 0.1 M H_2_SO_4_ (dashed line) and H_2_SO_4_+30 mM BZH (solid line), at 10 mV/s scan rate in 3 cycles. (b) CVs collected for Fe@C:N in 0.1 M H_2_SO_4_+1, 10, 30 mM BZH, at 10 mV/s scan rate in 3 cycles. Only cathodic region of last cycle is plotted.

The same catalyst ink was then used to investigate BZH reductions via electrolysis experiments. Figure 6a shows an SEM image of a 1 cm^2^ CC electrode decorated with a uniform drop‐cast layer of the electrocatalyst ink. SEM‐EDX mapping of the Fe@C:N/CC electrode in Figures 6b–6d indicates a uniform distribution of the ink content over the CC area, with a low Fe intensity arising from metal centers and F signal arising from the Nafion binder. Figure 7a shows uncompensated linear sweep voltammograms (LSV) of bare CC and of Fe@C:N/CC (1 cm^2^) obtained in an H‐cell (Figure S2) at 10 mV s^−1^ in 0.1 M H_2_SO_4_. In the presence of 30 mM of BZH a clear cathodic peak at *ca*. −0.7 V is observed in good agreement with results obtained at drop‐cast disk electrodes. An additional cathodic peak is observed at −0.26 V for Fe@C:N/CC that is absent in the case of bare CC. Figure 7b shows LSV collected for Fe@C : N/CC at varying BZH concentrations of 1.0, 10.0 and 30.0 mM, at 10 mV s^−1^; results show that the cathodic peak at −0.26 V does not change current density as a function of concentration thus suggesting that this peak is likely dominated by surface redox processes. On the other hand, the peak at −0.8 V increases with BZH concentration and can be clearly ascribed to an organic reduction.

The LSV in Figure 7a show an increased background current density for Fe@C:N/CC electrodes relative to bare CC. This was further investigated by measuring CV at varying scan rates in a non‐faradaic potential window, as shown in Figure S13. The double‐layer capacitance (C_dl_) was estimated from a linear fit of the capacitive currents and found to be 1.32 and 5.57 mF cm^−2^ for bare CC and Fe@C:N/CC, respectively. Therefore, drop‐casting of the catalyst ink increases the electrochemical specific surface area (ECSA); assuming the specific double‐layer capacitance to remain constant,[^56^, ^59^] the microscopic area can be estimated to increase approximately four‐fold after deposition of Fe@C:N/C on the CC support. This higher value is consistent with high porosity developing during the synthesis of the catalyst material.

**Figure 8:**
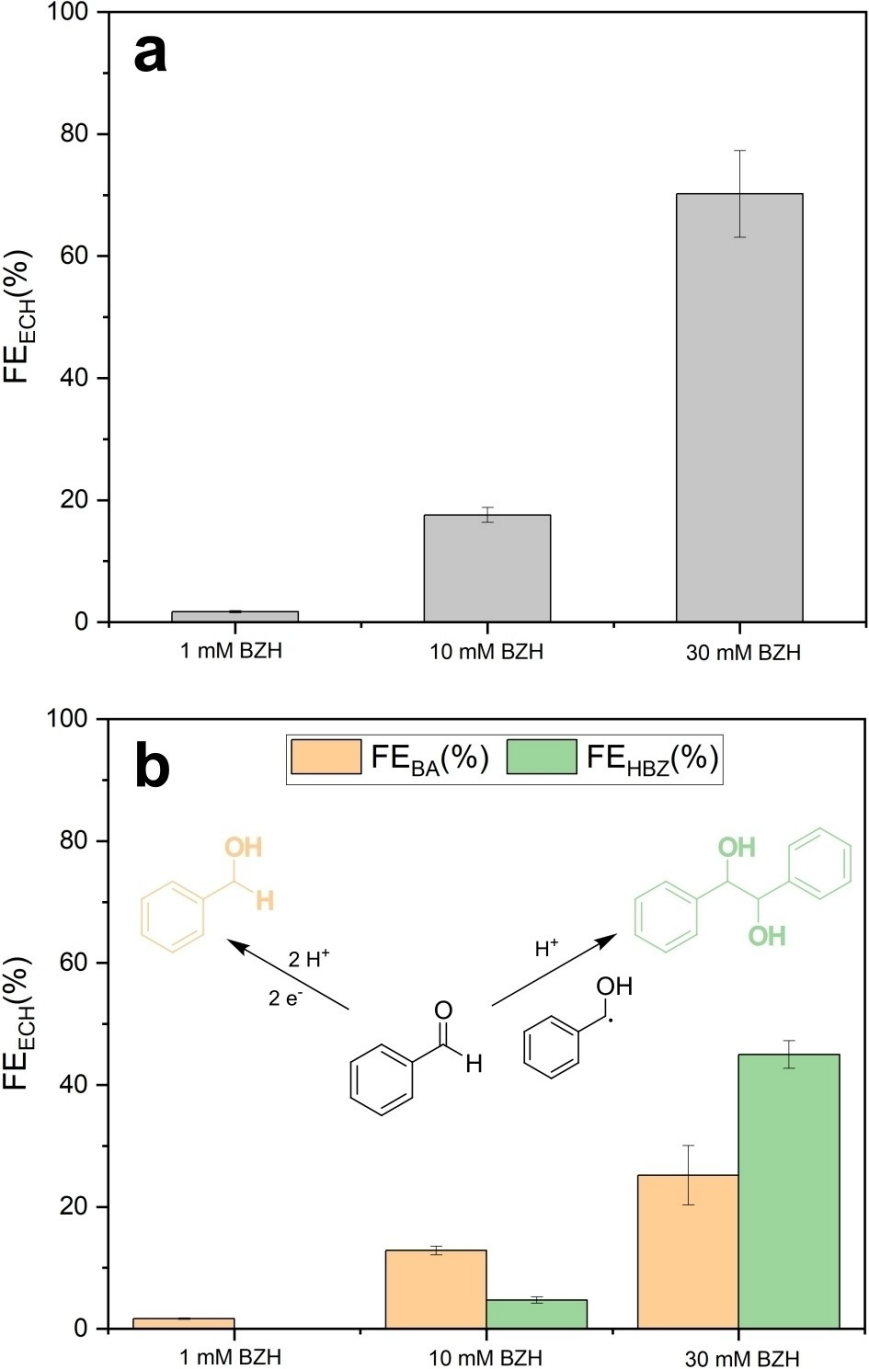
(a) Total faradaic efficiency (FE) for organic reductions at −0.8 V. (b) Contributions of benzyl alcohol (BA) and hydrobenzoin (HBZ) to the total faradaic efficiency of BZH reductions.

To further evaluate reactions taking place at cathodic potentials, electrolysis experiments with product analysis were carried out using the H‐cell setup described above. In a first set of experiments, chronoamperometry experiments were carried out at the peak potential of −0.8 V in 0.1 M H_2_SO_4_ solutions containing three concentrations of BZH for 2 h under static conditions. The current density remained stable over the entire duration of the experiment, thus confirming that the electrodes prepared as in Figure 6 display high stability even when subject to the mechanical stresses associated with evolution of hydrogen bubbles in the HER region (see Figure S14). Two products were identified from the chromatogram at t_2h_: benzyl alcohol (BA) and hydrobenzoin (HBZ), thus confirming that hydrogenation of the organic substrate takes place at the peak potential (Figure S15). For all the experiments the carbon balance was ca. 90 % (Figure S16), which supports that the majority of the products are being detected in the liquid phase of the catholyte compartment. Figure 8a shows the total faradaic efficiency (FE) associated with BZH reductions obtained as a function of BZH concentration. FE values were found to increase up to 70.1 % at 30.0 mM concentrations; higher concentrations were not tested to remain well below the solubility limit of the reactant (65 mM).^[36]^ It is important to note that although a cathodic peak can be observed at bare CC electrodes, the hydrogenation performance summarized in Figure 8 can be largely attributed to the presence of Fe@C:N; in fact, a control experiment (Figure S17) shows that the bare support yields less than half of the FE at Fe@C:N. Yield, selectivity and conversion values were also calculated and are shown in Supporting Information (Figure S18). The total yield for the reduction (Yield_ECH_%) decreases from 16.3 % to 7.2 % when the BZH concentration is increased from 1 to 30 mM. Similarly, BZH conversion decreases from 33.6 % to 24.3 % as the concentration of the benzaldehyde increases. These values are higher than estimates for a diffusion‐controlled reaction at a smooth 1 cm^2^ electrode with a similar cell and electrode geometry as in our experiments (Figure S19), thus suggesting that the reduction rates are facilitated by convection due to the generation of H_2_ bubbles, as previously reported.^[88]^ Further, under these electrolysis conditions the selectivity towards BA is highest at the lowest BZH concentrations, whereas the dimer becomes the preferred product at the highest concentration investigated. This suggests that when the BZH concentration increases from 1 to 30 mM the cathodic process results in increased ketyl radical concentrations leading to higher probability of dimerization via pinacolic coupling.^[89]^ On the other hand, at 1 mM the ketyl radicals should have a higher probability of reacting with protons or H_ads_ species at the electrode surface thus enhancing the selectivity towards BA.

**Figure 9:**
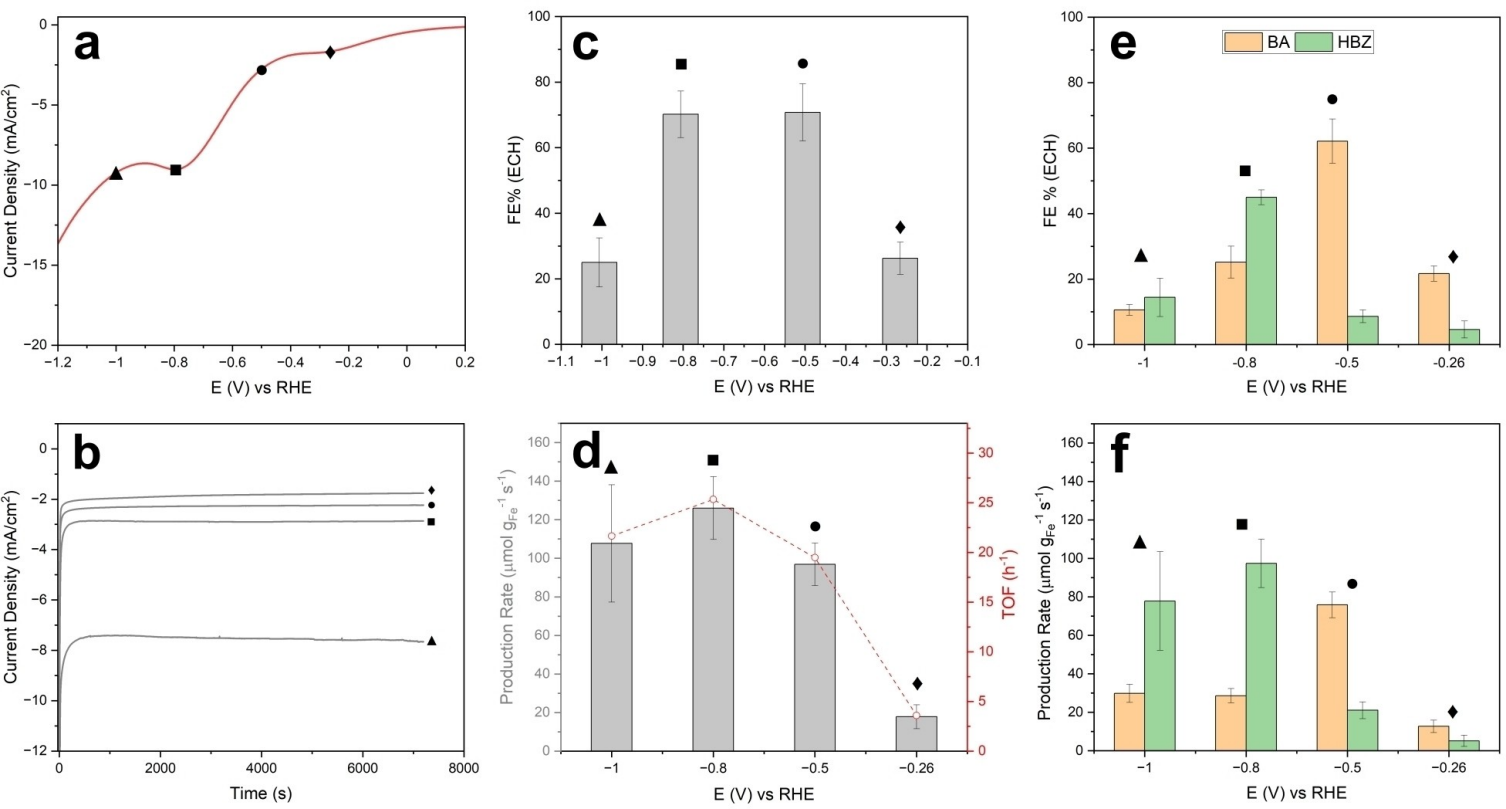
(a) CV collected for Fe@C:N on CC–MPL in 0.1 M H_2_SO_4_/30 mM BZH at 10 mV/s. (b) Chronoamperometries collected for each potential for 2 h. Total ECH FE % (c) and total product rate with TOF values (d) for −0.26, 0.5, −0.8, −1 V vs RHE. Contribution of BA and HBZ to ECH FE % (e) and to product rate (f) for each potential.

To better understand the interplay among product selectivity, FE and potential, we then carried out a more detailed investigation of hydrogenation performance indicators using the electrolyte composition that yielded the highest FE performance in Figure 7, i. e. 30 mM BZH in 0.1 M H_2_SO_4_. Figure 9a shows the uncompensated CV of a Fe@C:N/CC electrode in the above electrolyte, obtained in the H‐cell, with potentials marked at −0.26, −0.50, −0.80 and −1.00 V to indicate values chosen for further chronoamperometry experiments. The corresponding chronoamperograms are shown in Figure 9b; all plots display nearly constant currents, which is diagnostic of a highly stable electrode performance over the entire duration of the experiments. Figure 9c shows the change in total FE of the organic hydrogenations as a function of potential. First, it is interesting to note that there is detectable organic hydrogenation activity at potentials as high as −0.26 V vs RHE. A control experiment run under identical conditions but at open circuit confirmed that the products observed are indeed the result of an electrochemical hydrogenation process (see Figure S20). As the potential is lowered, the FE first increases and then decreases significantly at −1.00 V; in contrast, the average rate and TOF values for the production of hydrogenated organics remain approximately constant, as shown in Figure 9 d. These results are consistent with improvements in hydrogenation efficiency at −0.50 and −0.80 V, and the drop at −1.00 V being attributable to competition from increased rates of HER.^[90]^ Therefore, for potentials below −0.80 V, any further increases in cathodic current are almost exclusively due to increased rates of HER rather than to enhanced rates of organic hydrogenation. Finally, a control experiment using a bare CC in 30 mM BZH/0.1 M H_2_SO_4_ (Figure S21) further confirms that the faradaic efficiency at −0.50 V results from an improvement of more than a factor of two in the intrinsic efficiency of Fe@C:N surfaces towards ECH relative to that of a graphitic carbon support.

The trends in FE vs potential for the two products, BA and HBZ, are particularly interesting and are shown in Figure 9e. It is clear that under the conditions of our experiments, BA is the preferred product at more anodic potentials whereas HBZ is instead favored at the more cathodic potentials. This is also evident by examining the rates of production for the two compounds, shown in Figure 9f; the corresponding TOF values are reported in Supporting Information (Table S2). Notably, at −0.56 V the selectivity towards BA is very high, while also achieving a close to maximum average rate of production of hydrogenated organics and high total FE. This suggests that it is possible to tailor the electrolysis conditions to achieve electrochemical hydrogenations with high selectivity and high efficiency. Optimal conditions are likely to be found at intermediate potentials that are sufficiently cathodic to result in H_ads_ coverage,[^5^, ^91^] but also sufficiently anodic to inhibit uncontrolled/too fast reduction rates of BZH to α‐hydroxybenzyl radicals and, consequently, fast dimerization. In fact, greater selectivity for BA in Figure 9 is achieved at the lowest overpotentials relative to the thermodynamic value for the conversion of BZH to BA (E°=0.14–0.19 V).[^6^, ^92^] A similar mechanism for increased selectivity towards dimerization at base metals was proposed by Andrews et al.^[15]^ based on product trends in continuous flow cell experiments. Our data therefore suggests that using metal‐encapsulated Fe@C:N heterostructured materials it is possible to achieve hydrogenation via an electrocatalytic mechanism, as opposed to a direct electronation‐protonation pathway. This can take place over a selected potential range that enables fast rates of hydrogenation via surface‐activated proton transfer. It is important to note that the specific influence of nitrides and carbides on the ECH performances cannot be easily discriminated/resolved and fundamental studies on pure carbides and nitrides would be desirable in order to clarify this aspect.

Prior work on the reduction of BZH indicates that achieving high FE values while controlling selectivity for BA and/or HBZ remains a challenge in the literature, particularly when balanced against the choice of metal in terms of their cost and abundance.[^11^, ^12^, ^15^, ^19^, ^20^, ^87^] Electrode materials with high HER activity, such as Pt/C, catalyze hydrogenation selectively to BA at low overpotentials; however, as the potential is shifted to more cathodic values, competition from the HER lowers the FE of hydrogenations.^[20]^ This is similar to our observed trends in Figure 9e, further supporting the idea that BA production at Fe@C:N surfaces and low overpotentials takes place via H_ads_ transfers. It is interesting to note that FE values for Fe@C:N in this work at −0.26 V are only slightly inferior to those for Rh/C and Pt/C in pH 5 batch cells at similar potentials (vs. RHE) reported by Song et al., while the performance appears comparable or better at −0.50 V.^[20]^ TOF values at Fe@C:N are approximately two orders of magnitude lower than those reported for Rh/C and Pt/C in the same work, as expected from the much higher HER activity at these Pt‐group metals compared to Fe.^[83]^ However, using a continuous flow cell configuration with carbon felt electrodes, Lopez‐Ruiz et al.^[93]^ reported TOF_ECH_ values at pH 2 and 5 mA cm^−2^ using Pd/C, one of the best metal electrodes for ECH of BZH, that are only ~12 times larger than those observed with Fe@C:N/CC at similar current density. Anibal et al.^[14]^ also investigated BZH reduction studies on metal foils in pH 4.6 electrolyte at −0.5 V vs RHE; interestingly, rates of production at −0.56 V with Fe@C:N in this work are intermediate between those reported in the study for Pd and Pt, and are similar to those measured at Au foils. Given the well‐known difference in cost and criticality between Fe and Pt‐group metals, the performances observed at Fe@C:N appear promising to further develop novel Fe‐based composite electrocatalysts with selectivity towards the alcohol.

ECH of BZH using base metal electrodes has been previously investigated and the performance of Fe@C:N compares very well to those of e. g. Ni^[20]^ and Cu^[14]^ in terms of TOF values and production rates in aqueous electrolytes. To the best of our knowledge there are no reports of ECH of BZH at Fe or Fe@C electrodes, however there have been several studies using iron electrodes for the reduction of other aldehydes. In such previous work, high selectivity towards the simple alcohol product is not usually observed.[^23^, ^25^, ^29^, ^30^] For instance, in acid electrolyte the ECH of furfural using iron electrodes has been shown to lead almost exclusively to the pinacolic dimer^[29]^ or to the highly hydrogenated methylfuran product.^[30]^ Hydrogenation of glucose to sorbitol is somewhat of an exception, as it was observed to take place with high selectivity using Fe at neutral pH, but no hydrogenated products were detected in sulfuric acid solutions, as the hydrogenation was proposed to require a local alkaline pH to proceed.^[25]^ Mixed product streams with different degree of hydrogenation were detected at Fe electrodes using HMF as a substrate in acid electrolyte.^[23]^ Under our experimental conditions, we observed BA with good selectivity at intermediate potentials and neither hydrogenation of the aromatic ring or hydrogenolysis to toluene were detected. The BA‐selectivity is likely due to a combination of higher stability of phenyl vs. furanyl rings and differences in the organic‐surface adsorption strengths. Although this argument is consistent with descriptors and reactivity trends observed in the literature,[^90^, ^94^] further studies aimed at modulating the partitioning of BZH onto the surface are needed to support this hypothesis. An interesting aspect of the Fe@C : N electrocatalyst architecture is that the rich library of surface functionalization strategies applicable to carbon nanomaterials is available to fine tune the binding strengths of substrate and intermediates, as previously demonstrated in our group for other organic and inorganic redox processes.[^55^, ^80^, ^95^]

## 4 Conclusions

In this work we prepared and studied a porous Fe@C:N heterostructured material via hydrothermal synthesis. We introduced a new annealing protocol that includes a pre‐annealing step and that yields predominantly Fe/Fe_x_C metal sites at the carbon surface with only small FeO_x_ content, in contrast with previously reported methods.^[33]^ To evaluate the performance of the material as electrocatalyst in organic hydrogenations we used benzaldehyde as a diagnostic substrate. Chronoamperometry coupled with product analysis using gas chromatography demonstrate that it is possible to achieve hydrogenation of benzaldehyde to the alcohol and the pinacolic coupling products.

Interestingly, results show that the applied potential can influence the contribution to the total faradaic efficiency for hydrogenations of the two products, thus indicating that electrolysis conditions can be tailored to achieve both high product selectivity and high efficiency. Optimal conditions were identified at intermediate potentials that enable control over the competition between ECH and HER, with BA and HBZ being the preferred products at low and high overpotentials, respectively. Estimates of TOF and production rates compare extremely well to those reported using base metal electrodes such Ni and Cu for the ECH of BZH in aqueous electrolytes.[^14^, ^20^] Notably, TOF values for Fe@C:N are only an order of magnitude smaller than those observed for a carbon supported precious metal electrocatalysts such as Pd/C (pH 2) in previous reports.^[20]^ Given that our experiments are not carried out under conditions that necessarily maximize mass transport, the calculated TOF values for Fe@C: N are likely to represent a lower boundary value.

To the best of our knowledge, there are no reports of comparable or better performances in the ECH of benzaldehyde using iron‐carbon composites. Considering the wide possibility of implementing these architectures with different functionalizations, we believe that our results could open the door to improved M@C:N architectures for hydrogenation of organics using low‐cost and abundant metals not yet explored in this configuration and tested as catalysts for different organic substrates.

## Conflict of Interests

The authors declare no conflict of interest.

5

## Supporting information

As a service to our authors and readers, this journal provides supporting information supplied by the authors. Such materials are peer reviewed and may be re‐organized for online delivery, but are not copy‐edited or typeset. Technical support issues arising from supporting information (other than missing files) should be addressed to the authors.

Supporting Information

## Data Availability

The data that support the findings of this study are available from the corresponding author upon reasonable request.
