# Supplementary material for: Porous N‐Doped Carbon‐encapsulated Iron as Novel Catalyst Architecture for the Electrocatalytic Hydrogenation of Benzaldehyde
Source: ChemSusChem. 2024 Oct 17;18(1):e202400546. doi: 10.1002/cssc.202400546 (PMC11696197; doi:10.1002/cssc.202400546)
Supplement: Supplementary file 1 — Supporting Information [file CSSC-18-e202400546-s001.pdf]

ChemSusChem

Supporting Information

Porous N-Doped Carbon-encapsulated Iron as Novel Catalyst Architecture for the Electrocatalytic Hydrogenation of Benzaldehyde

Filippo Pota, Maida A. Costa de Oliveira, Christian Schröder, Marc Brunet Cabré, Hugo Nolan, Aran Rafferty, Olivier Jeannin, Franck Camerel, James A. Behan, Frédéric Barrière, and Paula E. Colavita*

Supporting Information for

Porous N-doped carbon-encapsulated iron as novel catalyst architecture for the electrocatalytic hydrogenation of benzaldehyde

Filippo Pota,[1] Maida Aysla Costa de Oliveira,[1] Christian Schröder,[1] Marc Brunet Cabré,[1] Hugo Nolan,[1] Aran Rafferty,[1] Olivier Jeannin,[2] Franck Camerel,[2] James A. Behan,[2] Frédéric Barrière,[2] Paula E. Colavita[1]*

1 - School of Chemistry, Trinity College Dublin, College Green, Dublin 2, Ireland

2 - Univ Rennes, CNRS, Institut des Sciences Chimiques de Rennes – UMR 6226, F-35000 Rennes, France.

[*]Corresponding author: colavitp@tcd.ie

List of Supporting Information Contents

Figure S1: Annealing procedure

Figure S2: H-cell scheme

Figure S3: N_2 adsorption isotherm and pore distribution

Figure S4: SAXS intensity plot.

Figure S5: FT-IR spectrum

Figure S6: Raman for FeRPM and Fe@C:N

Figure S7: Fe^0 XRD pattern

Figure S8: XPS O 1s spectrum

FigureS9: XPS C 1s high resolution spectra for FeRPM and Fe@C:N

Figure S10: SEM Fe@C:N

Figure S11: HER with Pt electrode

Figure S12: Tafel slope

Table S1: Overpot, Tafel slope, exchange current

Figure S13: Capacitance measures

Figure S14: Chronoamperometry experiments and iR drop measures (1, 10, 30 mM)

Figure S15: Gas Chromatography of BA and HBZ for 30 mM BZH

Figure S16: Mass balance

Figure S17: Control experiment Bare CC for FE and Yields

Figure S18: Yield, Selectivity and Conversion (1, 10, 30 mM)

Figure S19: Estimation of diffusive current in H-cell geometry by FEA

Table S2: FEA simulation boundary conditions

Table S3: FEA simulation parameters

Figure S20: Open circuit experiment Gas Chromatography

Table S4: Summary reaction data for ECH and HER

Figure S21: Control experiment using Bare CC at -0.5 V.

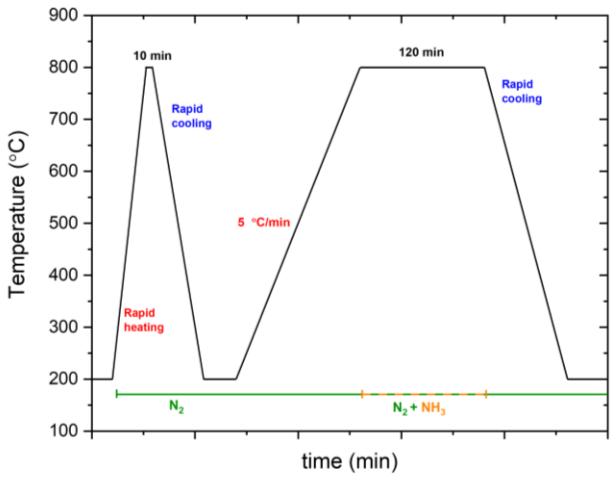

Figure S1. Annealing procedure T vs time.

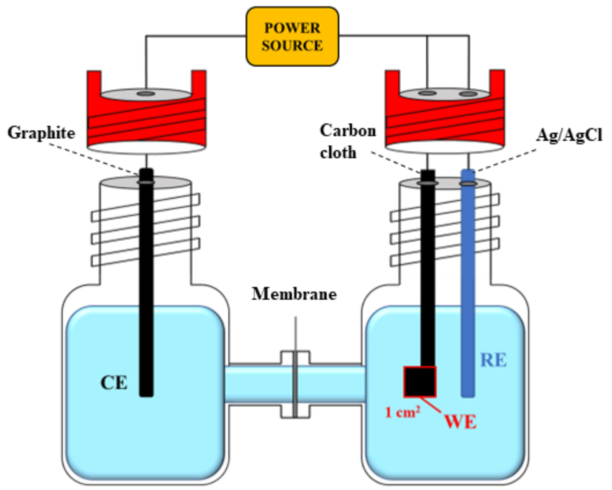

Figure S2. Schematic representation of the H-cell used for electrolysis experiments.

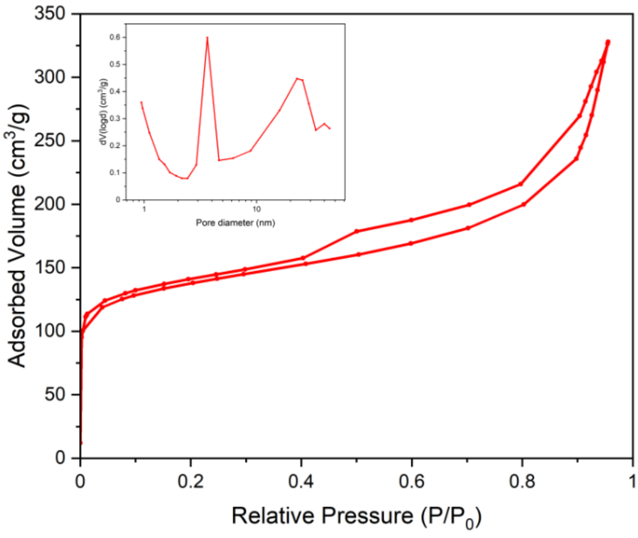

Figure S3. Nitrogen adsorption-desorption isotherm and pore size distribution (inset).

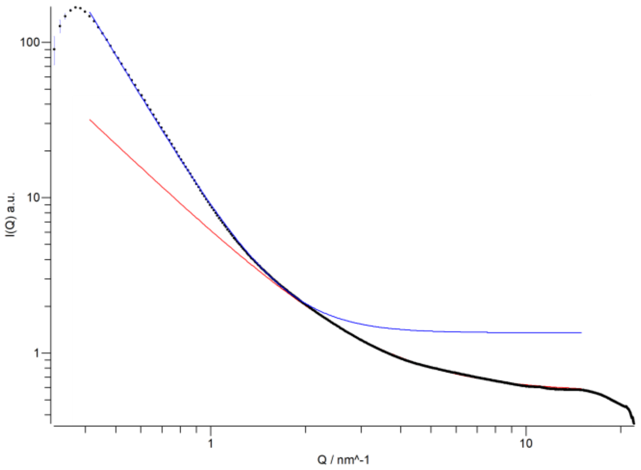

Figure S4. Log-log plot and best-fits of corrected SAXS intensity (arbitrary units) vs q (nm^{-1}). 15 one-hour images of the capillary and sample were collected and processed using Fit2D; then data were corrected from absorption and the contribution of the empty capillary was subtracted. The pattern shows no diffraction peaks in the explored q range, thus indicating the absence of pore mesostructures. Fits were carried out using SASfit (v.0.94.12) using two power laws of general formulae $I = C_0 + C_1 q^{-\alpha}$. In the range 10 – 2 nm^{-1}, the intensity follows a q^{-2} law (red trace), which can be attributed to graphitic layers in the sample. Below 2 nm^{-1}, the intensity follows a $q^{-3.4}$ dependence, corresponding to a deviated Porod law (blue trace). Deviations from Porod law can be attributed to a rough interface between the graphitic matrix and the pores.

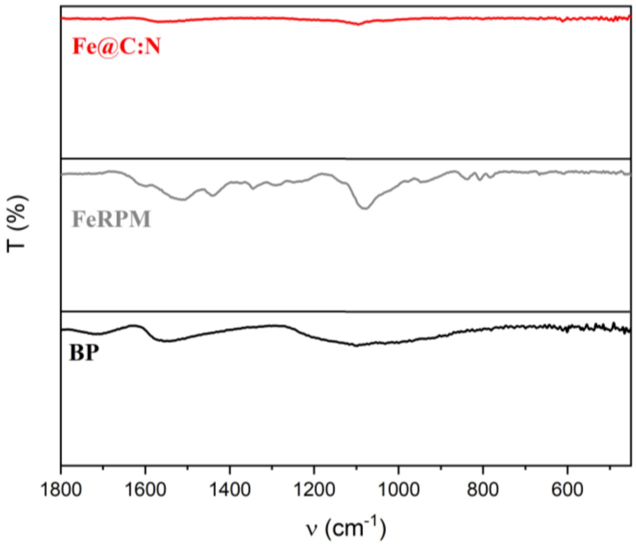

Figure S5. FTIR spectrum collected for Pure BP, FeRPM (pre-annealing) and Fe@C:N.

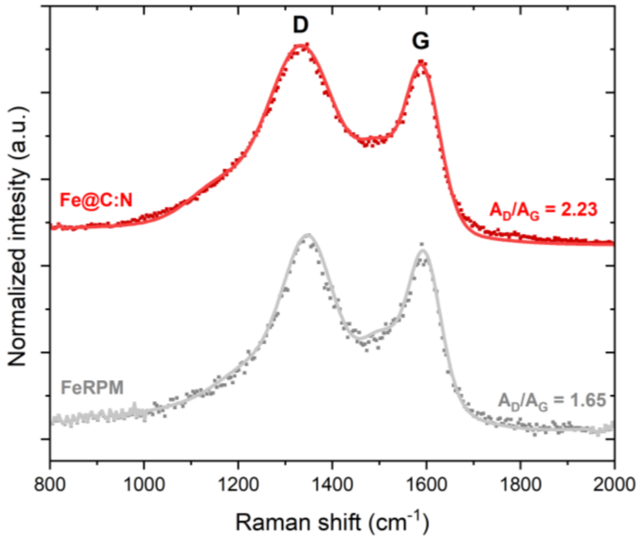

Figure S6. Raman spectra and best-fits of FeRPM and Fe@C:N; spectra are shown , normalised by the G-peak height to facilitate comparison.

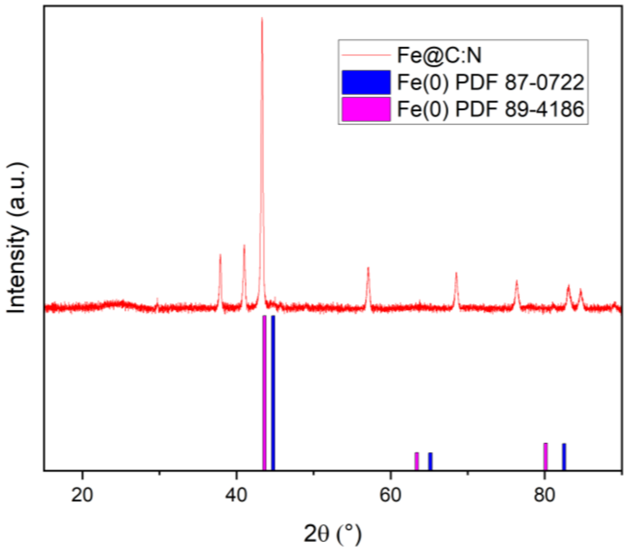

Figure S7. XRD patterns of Fe@C:N compared to reference patterns of Fe^0.

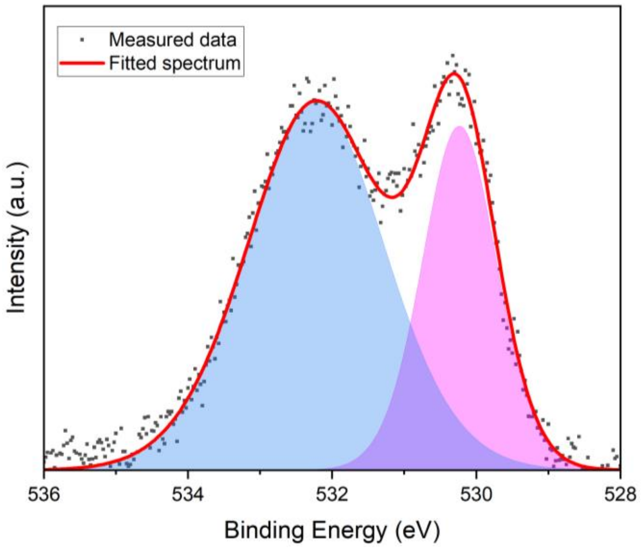

Figure S8. O 1s high resolution spectrum and best fit with components attributed to Fe@C:N and In_2O_3.[1]

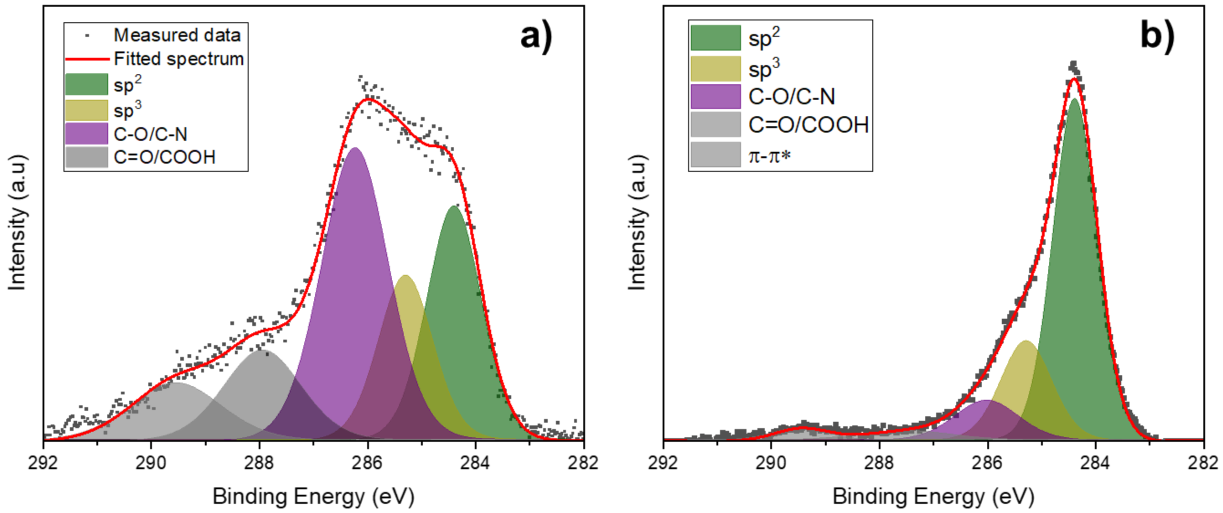

Figure S9. Comparison of C 1s high resolution spectra and best-fits of (a) FeRPM and (b) Fe@C:N.

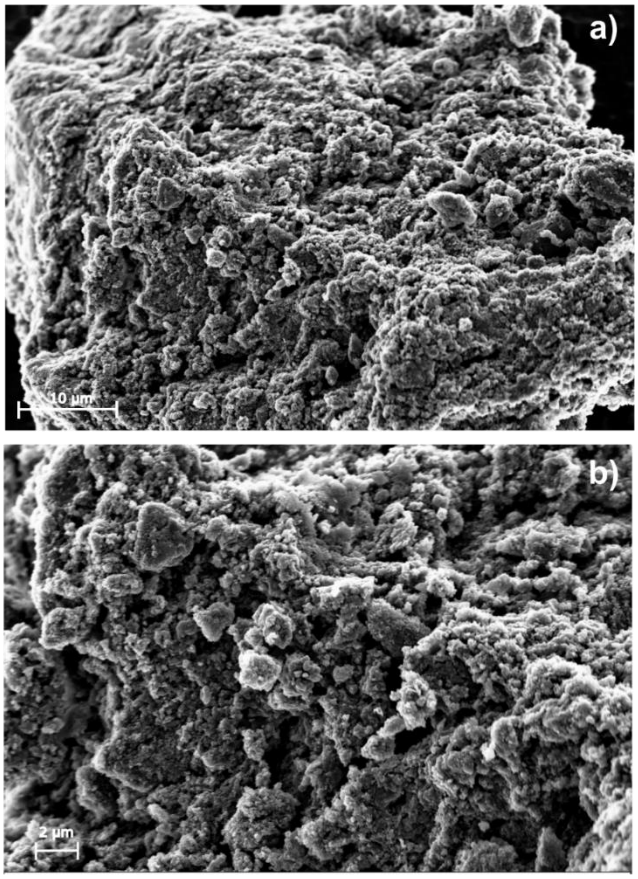

Figure S10. SEM images of Fe@C:N.

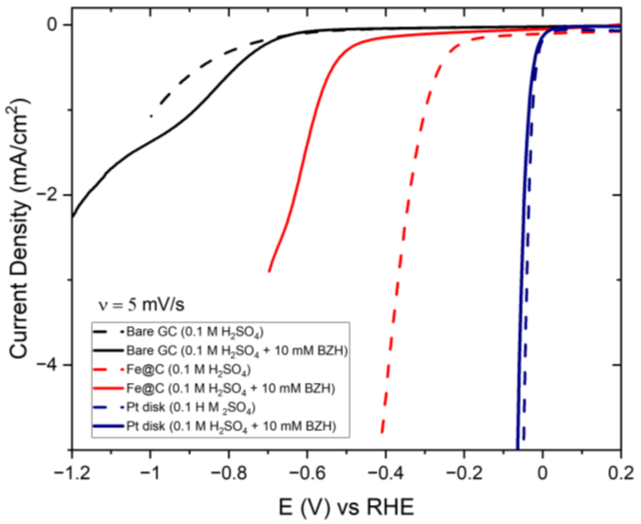

Figure S11. CVs collected for bare CC (black), Fe@C:N (red) and Pt disk (blue) in 0.1 M H_2SO_4 (dash line) and 0.1 M H_2SO_4 with 10 mM BZH (solid line), at 5 mV/s scan rate in 3 cycles. Only cathodic region of last cycle is plotted.

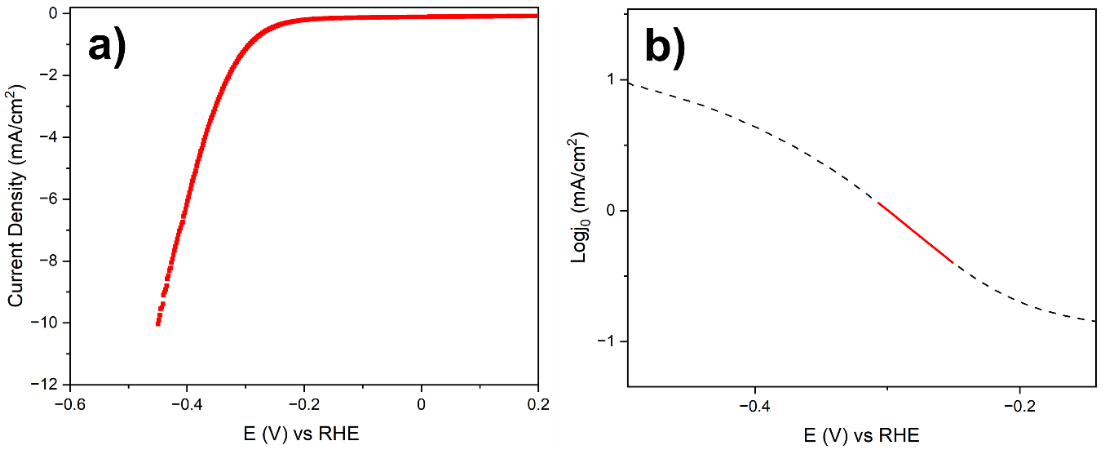

Figure S12. (a) LSV iR corrected for Fe@C:N in 0.1 M H_2SO_4 at 5 mV/s and (b) Tafel plot.

Table S1. Electrochemical performance indicators for Fe@C:N.

Material	Overpotential $\eta_{10\ mA}$ (V)	Tafel slope (mV/dec)	Exchange current density $\log j_0$ (mA/cm^2)
**Fe@C:N**	-0.45	123	-2.4

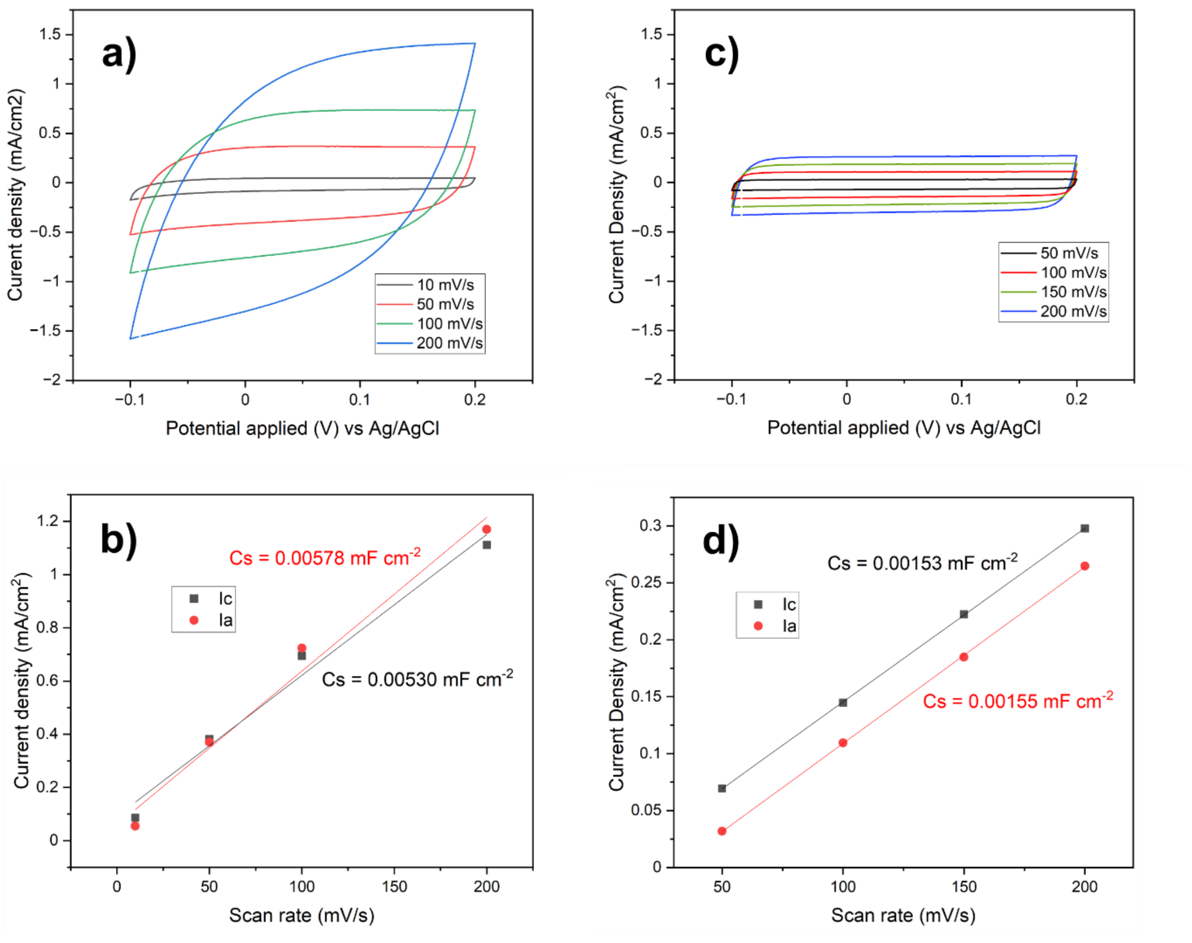

Figure S13. Non-Faraday CV profiles and current density plotted against the scan rate for (a, b) Fe@C:N and (c, d) Bare CC. Measurements were conducted in Na_2SO_4 0.1 M.

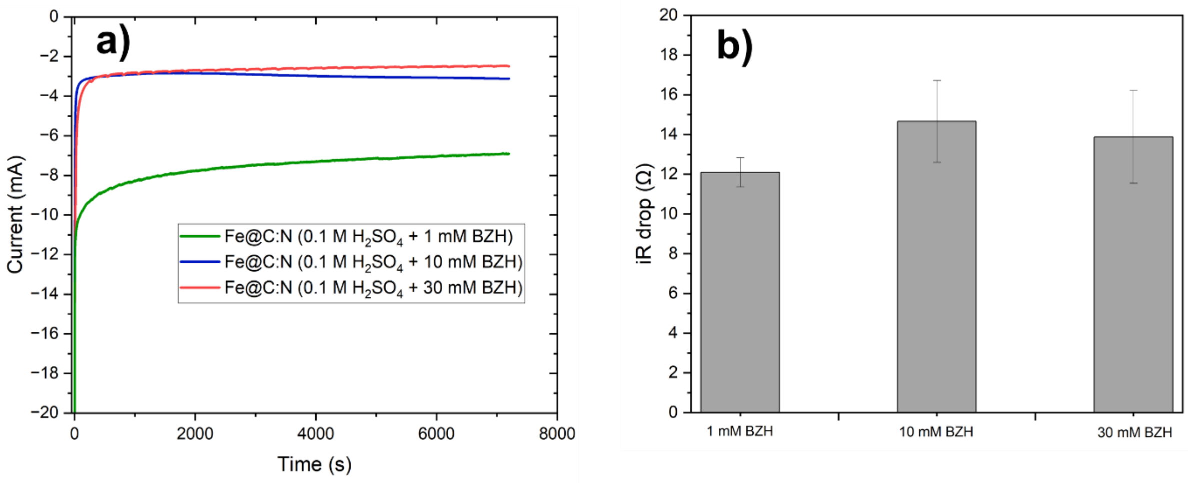

Figure S14. (a) Chronoamperometry and (b) iR drop measured for the electrodes in 1, 10 and 30 mM BZH.

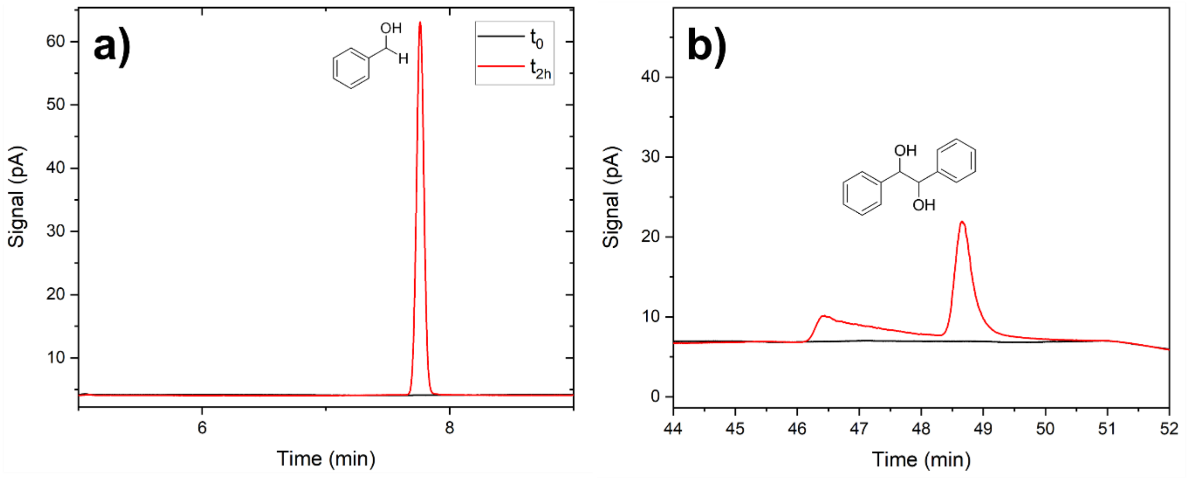

Figure S15. Overlay of t_0 and t_{2h} gas chromatograms for (a) BA and (b) HBZ in 0.1 M H_2SO_4 with 30 mM BZH.

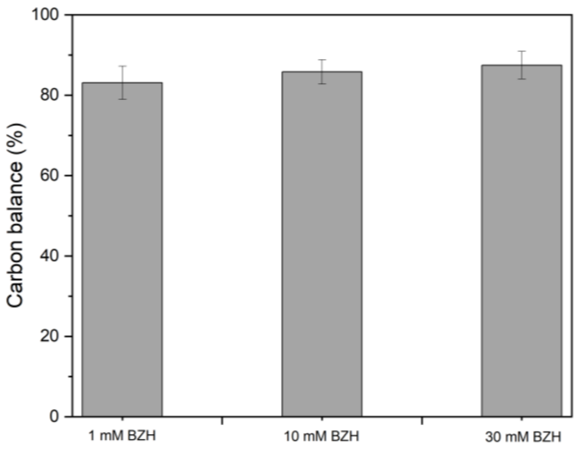

Figure S16. Carbon balance for 1, 10, and 30 mM BZH in 0.1 M H_2SO_4.

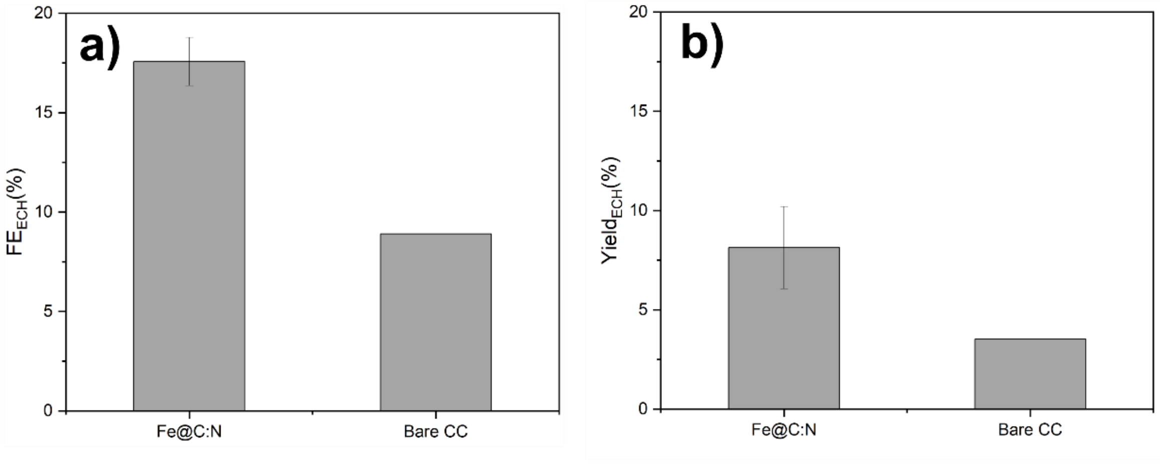

Figure S17. (a) FE_{ECH} and (b) $Yield_{ECH}$ of Fe@C:N and Bare CC obtained at -0.8 V vs RHE for 2 h in 0.1 M H_2SO_4 with 10 mM BZH.

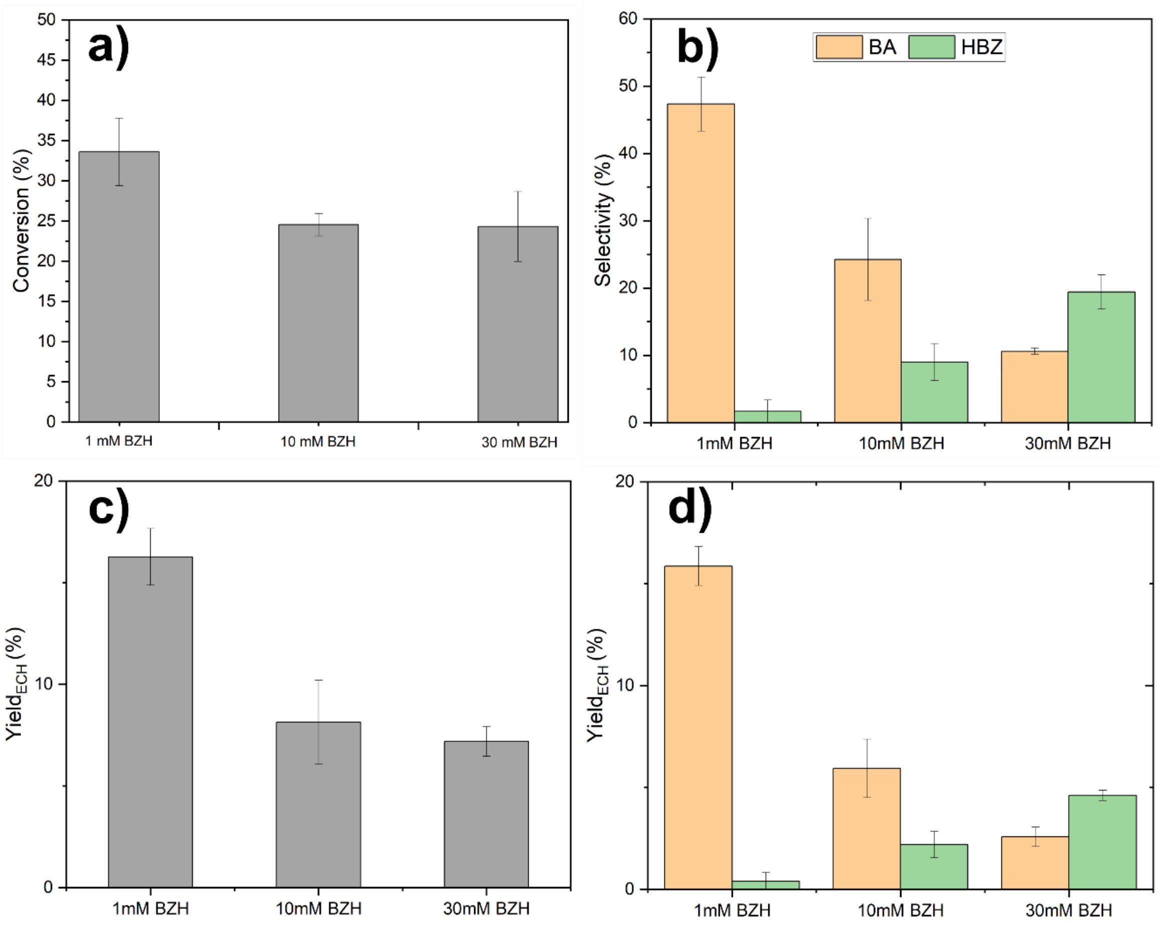

Figure S18. (a) Conversion, (b) BA and HBZ Selectivity, (c) total ECH Yield and (d) BA and HBZ Yield for 1, 10, 30 mM in 0.1 M H_2SO_4.

Estimation of diffusive current in H-cell geometry by Finite Element Analysis

Figure S19 provides the details of the model implemented to simulate diffusive mass transport through Finite Element Analysis (FEA) by COMSOL Multiphysics version 5.4. The model takes into account the diffusion coefficient of BZH, the specific geometry of the cell used and ensures the same volume of electrolyte as for experimental determinations (32 mL). A comprehensive description of the boundary conditions and parameters is provided in **Table S2** and **S3**. We found that the maximum conversion achievable solely through diffusive processes over a 2 hour period is 1.86 %, as illustrated in **Figure S19c**. Considering that mass losses typically observed at open circuit are only ca. 7.4% (see **Figure S20**), we conclude that the 24% conversion measured at 30 mM BZH (**Figure S18**) is much higher than values expected based on a diffusion-controlled reaction alone. Thus, convection e.g. due to gas evolution, must contribute significantly to the mass transport under our electrolysis conditions.

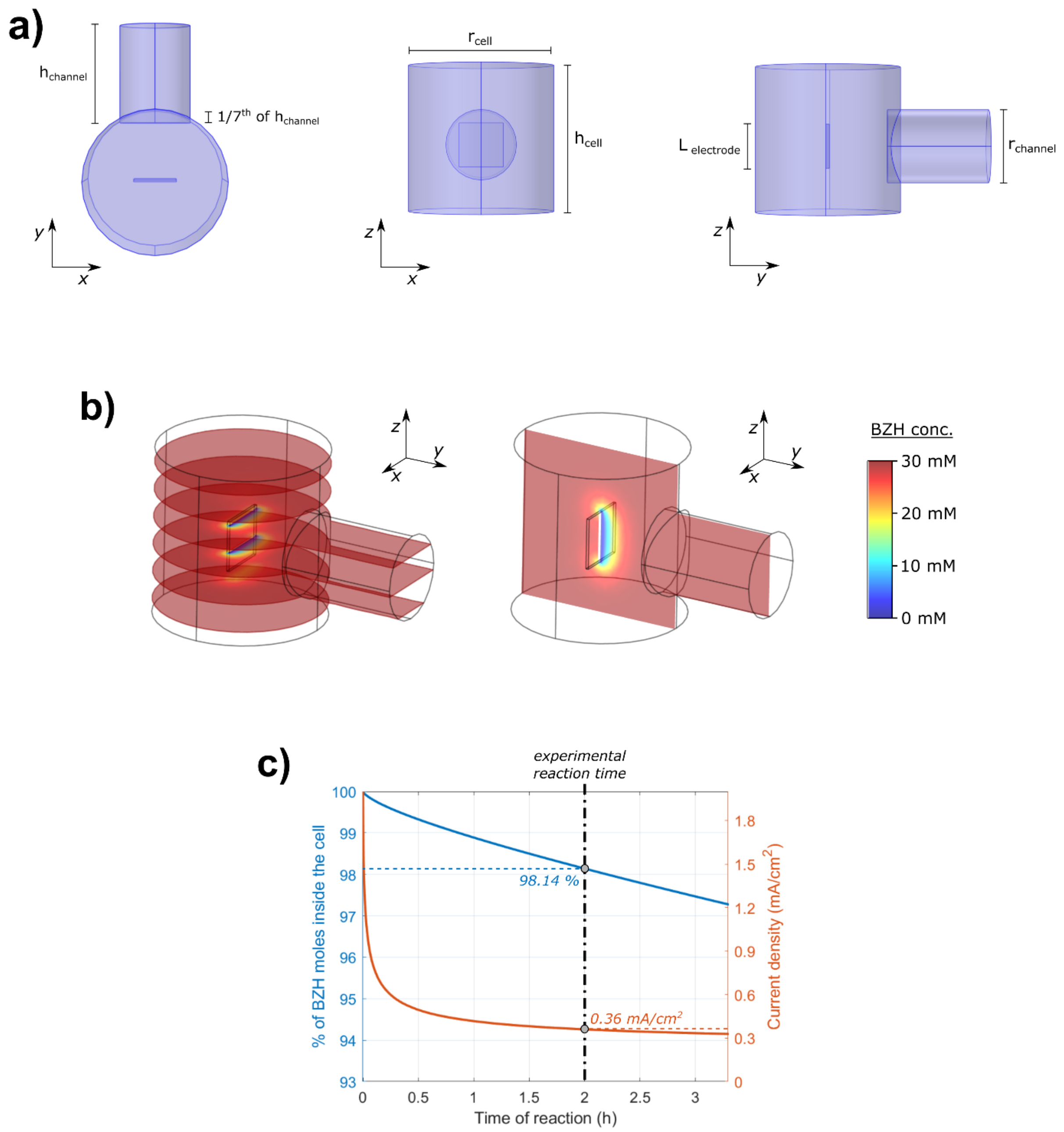

Figure S19. (a) Geometry of the cell simulated from different plane view perspectives with key geometric parameters identified. (b) Cell cross-sections profile showing BZH concentration on the cell after 2h reaction considering only diffusive mass transport. (c) On the left axis, evolution of the percentage of BZH moles remaining (from 30 mM BZH initial concentration) in the cell vs reaction time. On the right axis, evolution of current density vs reaction time. (a) and (b) are generated and exported directly from COMSOL Multiphysics.

Table S2. Coordinates (*z*,*y*,*z*) used to describe each boundary and domain in the finite element simulation as detailed in Fig. S14, and correspoding mass transport conditions. c_i refers to the concentration of i species.

**Boundary Description**		**Coordinates**	**Boundary condition**
Cell Domain	Top cap	$x^2 + y^2 \leq {r_{cell}}^2$ for $z = h_{cell}$	J = 0 (No flux)
	Bottom cap	$x^2 + y^2 \leq {r_{cell}}^2$ for $z = 0$	
	Cell wall	$x^2 + y^2 = {r_{cell}}^2$ for $0 \leq z \leq h_{cell}$	
Channel Domain	Cap	$z^2 + x^2 \leq {r_{channel}}^2$ for $y = r_{cell} + h_{channel} - \frac{h_{channel}}{7}$	J = 0 (No flux)
	Channel walls	$z^2 + x^2 = {r_{channel}}^2$ for $r_{cell} - \frac{h_{channel}}{7} \leq y \leq r_{cell} + h_{channel} - \frac{h_{channel}}{7}$	
Electrode Domain	Front Electrode Surface	$-\frac{1}{2}L_{electrode} \leq x \leq +\frac{1}{2}L_{electrode}$ $\frac{1}{2}h_{cell} - \frac{1}{2}L_{electrode} \leq z \leq \frac{1}{2}h_{cell} + \frac{1}{2}L_{electrode}$ $y = \frac{1}{2}d_{electrode}$	$c_{BZH} = 0$
	Back inert surface	$-\frac{1}{2}L_{electrode} \leq x \leq +\frac{1}{2}L_{electrode}$ $\frac{1}{2}h_{cell} - \frac{1}{2}L_{electrode} \leq z \leq \frac{1}{2}h_{cell} + \frac{1}{2}L_{electrode}$ $y = -\frac{1}{2}d_{electrode}$	J = 0 (No flux)
	Other inert electrode surface	The electrode has a thickness of $d_{electrode}$. There are 4 rectangular surfaces of size $d_{electrode}\ x\ L_{electrode}$ that enclose the Front and back surface of the electrode.	J = 0 (No flux)

Table S3. Summary of parameters used for finite element analysis.

Variable	Value	Units	Description
r_{cell}	16.5	mm	Radius of the cell domain.
h_{cell}	33	mm	Height of the cell domain.
$r_{channel}$	8.3	mm	Radius of the channel domain.
$h_{channel}$	23	mm	Height of the channel domain.
$L_{electrode}$	10	mm	Lateral length of the square electrode domain.
$d_{electrode}$	0.5	mm	Thickness of the electrode domain.
D_{BZH}	$8.6 \cdot 10^{-6}$	$cm^2\ s^{-1}$	Diffusion coefficient of Benzaldehyde[2]
$c0_{BZH}$	30	mM	Initial concentration of Benzaldehyde in the electrolyte solution domain. The electrode solution domain is contained within the cell and channel domain.

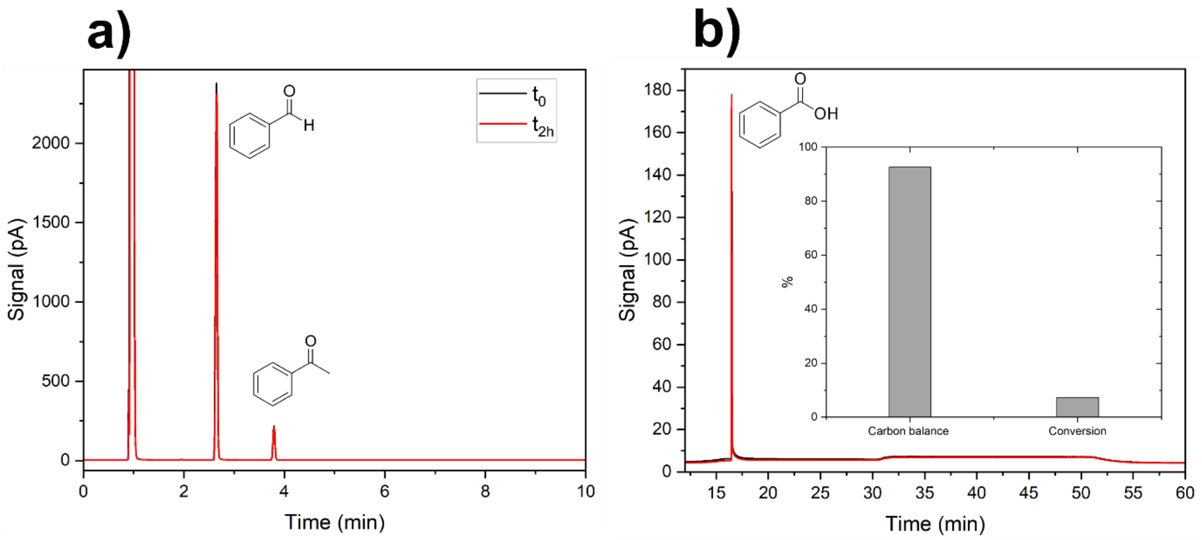

Figure S20. Overlay of t_0 and t_{2h} gas chromatograms collected in an open circuit control experiment from (a) 0 to 10 min and (b) 12 to 60 min.

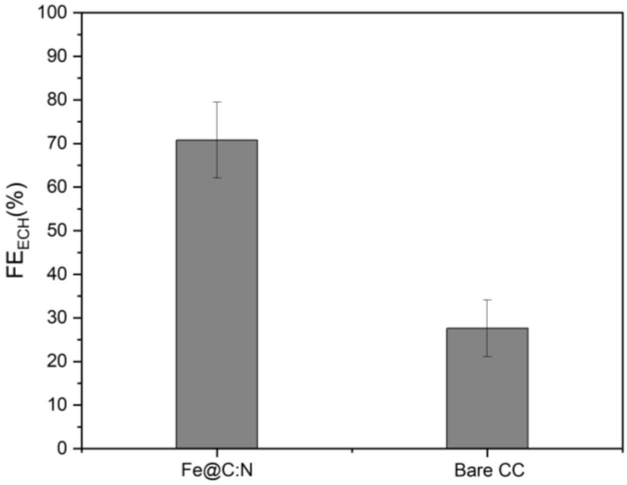

Figure S21. FE_{ECH} of Fe@C:N and bare CC obtained at -0.5 V vs RHE for 2 h in 0.1 M H_2SO_4 with 30 mM BZH.

Summary of equations

(1) $$\textbf{Carbon balance}\ (\%) = \frac{mol_{t_{2h}}(BZH) + mol_{t_{2h}}(BA) + 2mol_{t_{2h}}(HBZ)}{mol_{t_0}(BZH)} \cdot 100$$

(2) $$\textbf{Coversion}\ (\%) = \frac{mol_{t_0}\ (BZH) - mol_{t_{2h}}(BZH)}{mol_{t_0}(BZH)} \cdot 100$$

▾ (3) $$\textbf{Selectivity}_{\textbf{BA}}\ (\%) = \frac{mol_{t_{2h}}\ (BA)}{mol_{t_0}(BZH) - \ mol_{t_{2h}}(BZH)} \cdot 100$$

▾ (4) $$\textbf{Yield}_{\textbf{BA}}\ (\%) = \frac{mol_{t_{2h}}\ (BA)}{mol_{t_0}(BZH)} \cdot 100$$

▾ (5) $$\textbf{FE}_{\textbf{BA}}(\%) = \frac{2\ mol_{t_{2h}}\ (BA)}{mol_e} \cdot 100$$

(6) $$\textbf{FE}_{\textbf{ECH}}(\%) = \ FE_{BA} + FE_{HBZ}$$

(7) $$\textbf{FE}_{\textbf{HER}}(\%) = \ 100 - FE_{ECH}$$

(8) $$\textbf{Prod Rate}_{\textbf{BA}}(\mu mol\ g_{Fe}^{-1}\ s^{-1}) = \frac{n_{BA}}{M\ \cdot t}$$

(9) $$\textbf{Prod Rate}_{\textbf{HBZ}}(\mu mol\ g_{Fe}^{-1}\ s^{-1}) = \frac{n_{HBZ}}{M\ \cdot t} \times 2$$

(10) $$\textbf{Prod Rate}_{\textbf{HER}}(\mu mol\ g_{Fe}^{-1}\ s^{-1}) = \frac{n_e}{M\ \cdot t}$$

▾ (11) $$\textbf{TOF}_{\textbf{BA}}(h^{-1}) = \ Prod\ rate_{BA}(mol\ mg_{Fe}^{-1}\ h^{-1}\ ) \times MW_{Fe}\ (mol\ mg^{-1})$$

(12) $$\textbf{TOF}_{\textbf{ECH}}(h^{-1}) = TOF_{BA} + \ TOF_{HBZ}$$

▾ = Same calculation used for HBZ

Metal loading (M) = $1.2 \cdot 10^{-4}$ g/cm^2

Table S4. Summary reaction data for electrocatalytic hydrogenation (ECH) and hydrogen evolution reaction (HER), considering the single contribution of BA and HBZ.

E (V vs RHE)	mol e^- (μmol)	FE_{BA} (%)	FE_{HBZ} (%)	FE_{HER} (%)	Prod $Rate_{BA}$ (μmol g^{-1}_{Fe} s^{-1})	Prod $Rate_{HBZ}$ (μmol g^{-1}_{Fe} s^{-1})	Prod $Rate_{HER}$ (μmol g^{-1}_{Fe} s^{-1})	TOF_{BA} (h^{-1})	TOF_{HBZ} (h^{-1})	TOF_{ECH} (h^{-1})
-0.26	102 ± 29	22 ± 2	5 ± 3	73 ± 5	13 ± 3	5 ± 3	118 ± 34	2.5 ± 0.7	1.0 ± 0.6	3.5 ± 1.0
-0.50	214 ± 35	62 ± 7	9 ± 2	29 ± 7	76 ± 7	21 ± 4	248 ± 41	15 ± 1	4.2 ± 0.8	19 ± 1
-0.80	206 ± 8	25 ± 5	45 ± 2	30 ± 7	29 ± 4	97 ± 13	239 ± 9	5.7 ± 0.7	20 ± 3	25 ± 3
-1.00	490 ± 59	11 ± 2	15 ± 6	75 ± 7	30 ± 5	78 ± 26	567 ± 68	6 ± 1	16 ± 5	22 ± 5

References

[1] Z. M. Detweiler, S. M. Wulfsberg, M. G. Frith, A. B. Bocarsly, S. L. Bernasek, *Surface Science* **2016**, *648*, 188-195.

[2] J. Winkelmann, *Diffusion in gases, liquids and electrolytes*, Springer, **2007**.
